# Basic Notions and Innovations in Electrochemical Advanced Oxidation Processes for Water Treatment

**DOI:** 10.1002/smsc.70332

**Published:** 2026-07-13

**Authors:** Fida Tanos, Chaimaa Gomri, Clément Trellu, Mehmet A. Oturan, Shuaishuai Li, Minghua Zhou, Kehinde D. Jayeola, Omotayo A. Arotiba, Elisama Vieira dos Santos, Carlos A. Martínez‐Huitle, Mikhael Bechelany, Marc Cretin

**Affiliations:** ^1^ Institut Européen des Membranes, IEM, UMR 5635 Univ Montpellier Centre national de la recherche scientifique (CNRS) ENSCM Montpellier France; ^2^ Laboratoire Géomatériaux et Environnement Université Gustave Eiffel Marne‐la‐Vallée France; ^3^ MOE Key Laboratory of Pollution Processes and Environmental Criteria Tianjin Key Laboratory of Environmental Technology for Complex Trans‐Media Pollution Tianjin Advanced Water Treatment Technology International Joint Research Center College of Environmental Science and Engineering Nankai University Tianjin China; ^4^ Department of Chemical Sciences University of Johannesburg Johannesburg South Africa; ^5^ Centre for Nanomaterials Science Research University of Johannesburg Johannesburg South Africa; ^6^ Renewable Energies and Environmental Sustainability Research Group Institute of Chemistry Federal University of Rio Grande do Norte Natal Rio Grande do Norte Brazil

**Keywords:** anodic oxidation, electro‐Fenton, electrochemical advanced oxidation processes, emerging contaminants, nanomaterials, wastewater treatment, water scarcity

## Abstract

Natural water bodies are increasingly contaminated by synthetic organic compounds from industrial, agricultural, and domestic sources. Many of these compounds, for instance, pharmaceuticals, dyes, and pesticides, are chemically stable and cannot be efficiently eliminated by the classical wastewater treatment technologies, resulting in their persistence in surface and groundwater. Therefore, innovative strategies are required. Here, we reviewed electrochemical methods, such as anodic oxidation, electro‐Fenton, photo‐assisted catalysis, and electrochemical activation of persulfate and peroxymonosulfate, to remove recalcitrant organic pollutants from contaminated water. These methods generate reactive species that break down persistent contaminants. As the performance of these methods is affected by operational parameters, such as the type of electrode material, the applied current and the chemical composition of the solution, a fine‐tuning of these parameters can enhance the removal efficiency of pollutant from water. We also discuss the contribution of computational modeling methods to obtain mechanistic insights that are crucial for developing and optimizing electrochemical water treatment technologies.

## Graphical abstract

Electrochemical advanced oxidation processes effectively degrade persistent organic pollutants, turning polluted water into a clean resource. Through the use of reactive species, hybrid systems, and computational modeling these approaches optimize treatment and open the door to innovative, sustainable next‐generation water purification technologies.

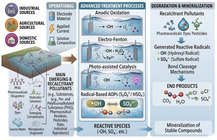

## 1 Introduction

Population growth, climate change, and industrialization explain the current high demands for water. On the other hand, water pollution, particularly by emerging contaminants, aggravates this problem [1]. As emerging, persistent contaminants are difficult to remove using traditional wastewater treatment methods, more effective and sustainable approaches are urgently required [2, 3, 4, 5, 6, 7].

In this context, electrochemical advanced oxidation processes represent an interesting alternative because they generate highly reactive oxygen species, particularly hydroxyl radicals (^•^OH), that break down recalcitrant pollutants into nontoxic byproducts. Moreover, these processes are cost‐effective, can be integrated in existing water treatment systems with minimal chemical inputs, and their performance can be optimized by acting on different operational parameters [8, 9, 10, 11, 12, 13]. However, the high cost of electrodes and the integration of renewable energy sources are two of the major challenges to their widespread utilization [14]. This review article offers a forward‐looking and application‐oriented description of electrochemical advanced oxidation processes, focusing on recently developed hybrid and sequential systems tailored for managing complex effluents. To address the challenge of recalcitrant pollutants, this review first establishes the fundamental and practical framework of anodic oxidation. Then, it evaluates its versatility in treating diverse contaminants, such as pharmaceuticals and per‐ and poly‐fluoroalkyl substances (PFAS), through direct electron transfer and hydroxyl radical‐mediated pathways. This section provides a practical roadmap for overcoming mass transfer and energy limitations through the use of reactive electrochemical membranes and optimized operational parameters for industrial scale‐up. Subsequently, the review evaluates the electro‐Fenton process, focusing on the in situ generation of the Fenton's reagent to eliminate the sludge production and chemical handling risks inherent in conventional Fenton chemistry. Specifically, it analyzes the practical transition to heterogeneous catalysts and activated carbon cathode regeneration, highlighting strategies for operating at near‐neutral pH and enhancing energy efficiency through preconcentration. In addition, photo‐electrooxidation is evaluated as a low‐energy anodic alternative. The review focuses on how bandgap engineering and morphology control improve visible‐light harvesting, and specifically analyzes S‐scheme and Z‐scheme heterojunctions as technical solutions to charge carrier recombination in solar‐driven systems. Furthermore, it assesses the emerging field of electrochemical persulfate and peroxymonosulfate activation, and compares anodic and cathodic pathways for the generation of sulfate radicals and singlet oxygen. This includes a practical examination of transition metal‐based anodes and three‐dimensional particle electrode systems as configurations to enhance nonradical oxidation. Finally, the review highlights the role of computational chemistry, specifically density functional theory (DFT) and electrical double layer modeling, in unraveling complex interfacial mechanisms at the reaction cage. By correlating theoretical simulations with experimental data, it demonstrates how these tools predict degradation pathways and enable the rational design of next‐generation electrodes while reducing experimental costs.

Table 1 presents a summary of some recent review articles on electrochemical advanced oxidation processes, highlighting the materials and techniques studied. It also shows the limitations and the aspects that were not addressed compared with the present review article. Indeed, this review stands out due to its a more detailed analysis of interfacial mechanisms and electrode performance for the removal of emerging pollutants.

**TABLE 1:** Comparison of this review and other high‐impact reviews in the field of electrochemical advanced oxidation processes from 2018–2025.

Topic/Aspect	This review	Other recent high‐impact reviews (2018–2025)	Key differences	References
Scope & technologies	Comprehensive coverage of anodic oxidation, electro‐Fenton, photo‐electrooxidation, persulfate activation, and computational chemistry.	Often focused on 1‐2 specific technologies (e.g., only electro‐Fenton, only persulfate activation) or a broader but less deep overview of electrochemical advanced oxidation processes.	Uniquely extensive and balanced, serving as a true “one‐stop” reference for multiple electrochemical advanced oxidation processes.	[15]
Computational chemistry	Dedicated, in‐depth section on the role of density functional theory, molecular modeling, and the “reaction cage” at the electrode–electrolyte interface.	Rarely covered in such depth. Most reviews mentioned it as a future perspective, but did not elaborate on mechanisms.	Pioneering focus on integrating theoretical and computational insights to explain fundamental mechanisms, a cutting‐edge approach.	[16]
Material innovation	Detailed discussion on novel anodes (e.g., Magnéli phases), cathodes and particle electrodes, including heterojunctions and doping for photo‐electrooxidation.	Focused on more conventional materials, boron‐doped diamond and mixed metal oxide or a specific class, (e.g., carbon‐based cathodes).	Greater emphasis on the latest material design strategies, particularly for complex systems, such as tandem and heterogeneous processes.	[17]
Per‐ and polyfluoroalkyl substances	Detailed mechanistic pathways provided for perfluorooctanoic acid/ perfluorooctanesulfonic acid degradation, including discussion on chain length and functional group effects.	Often covered as an application example without deep mechanistic insights.	Integrates a highly relevant and complex pollutant class with thorough chemical mechanism analysis in a broader electrochemical advanced oxidation processes context.	[18]
Process integration & scale‐up	Discusses integration with biological processes, activated carbon regeneration, and three‐dimensional reactor design, flow‐by vs. flow‐through.	Focused on laboratory‐scale performance metrics. Scale‐up and hybrid systems were often mentioned as challenges, not thoroughly described.	Bridges the gap between basic science, mechanisms and practical applications, reactor design, hybrid systems.	[19]

Many recent reviews provide an excellent overview of a specific technology (e.g., electro‐Fenton by Nidheesh et al. [16]) or a broader look at the entire field (e.g., Martínez‐Huitle et al. [15] and recent studies by Brillas and Garcia‐Segura [20] and Rim et al. [21]). On the other hand, the present review stands out by offering remarkable depth across a wider spectrum of electrochemical advanced oxidation processes. This review is also a significant and an original contribution because it devotes a section to computational methods, such as DFT and electrical double layer modeling, to unravel reaction mechanisms. This innovative approach has been rarely seen elsewhere. It moves beyond describing what works and starts to explain the underlying molecular mechanisms, which is crucial for the rational design of next‐generation electrodes and processes. Besides the standard boron‐doped diamond and mixed metal oxide anodes, this review extensively covers advanced materials, such as Magnéli phase (sub‐stoichiometric titanium oxide, (Ti_4_O_7_)), sophisticated heterojunction photoanodes, and engineered cathodes for heterogeneous Fenton catalysis, as novel and innovative materials.

Overall, this review bridges the gap between mechanistic understanding and practical applications, offering a holistic and forward‐looking perspective. It provides a unique resource for researchers and practitioners, clearly demonstrating its added value relative to prior reviews published between 2018 and 2025, and establishing a reference framework for designing next‐generation electrochemical water treatment processes.

## 2 Anodic Electrooxidation

**FIGURE 1:**
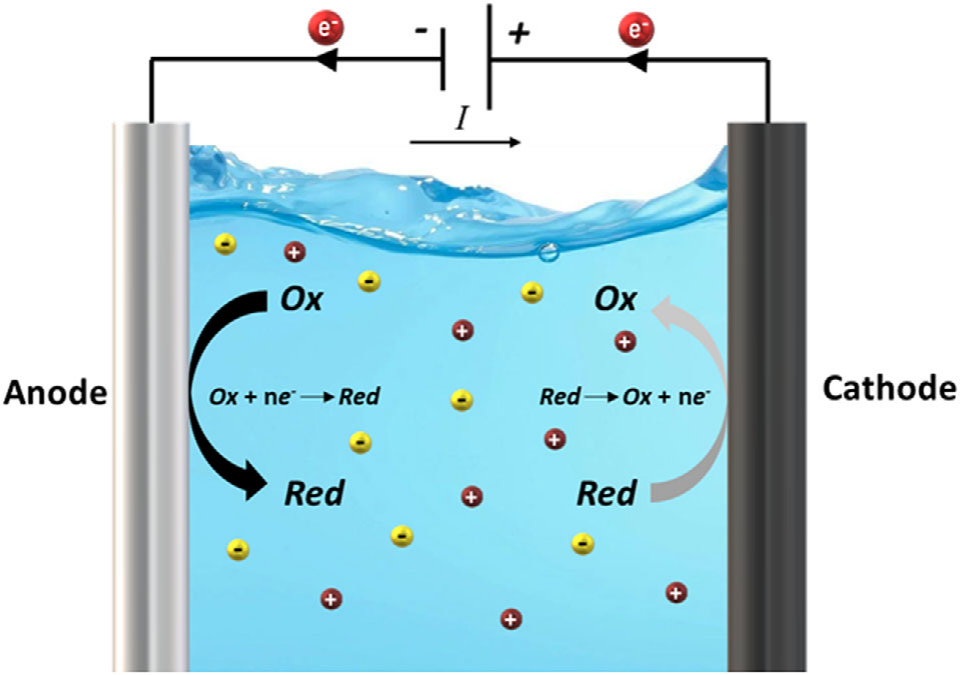
Electrolysis involves applying an electric current to drive the chemical reactions that degrade pollutants. The use of an electrocatalytic electrode enhances the oxidation reactions, breaking down contaminants into less harmful substances or completely mineralizing them. Electrooxidation is based on the direct oxidation at the electrode surface or indirect oxidation by the produced reactive species. Ox: oxidized species, Red: reduced species, e^−^: electron, *I*: electric current, ne^−^: number of electrons transferred in the redox reaction.

Anodic oxidation, also named electrooxidation, is an electrochemical advanced oxidation process for the degradation of recalcitrant pollutants in water. The main advantages of electrooxidation are: its versatility (it can be used to treat different contaminants at a large range of concentrations), eco‐friendliness (it is based on electrons as clean reagent), cost‐effectiveness, and simplicity of use [22, 23]. Electrooxidation is generally carried out in a nondivided electrolysis cell (Figure 1).

During electrooxidation, pollutants are oxidized by direct electron transfer or via oxidation through ^•^OH generated from water discharge at the anode surface [24]. The counter‐reaction at the cathode side can be water reduction, leading to hydrogen production and/or H_2_O_2_ production from O_2_ reduction during the electro‐Fenton process, as described in Section 3. The cathode acts as a counter‐electrode to allow the current circulation. Cathodes with good chemical and thermal stability are recommended, such as stainless steel, titanium, and carbon‐based material.

The ^•^OH produced at the anode surface according to Equation (1) is a powerful oxidizing agent, with high redox potential: 2.80 V versus a standard hydrogen electrode (SHE). ^•^OH is a highly reactive species and can attack organic compounds by eliminating hydrogen atoms or by addition reactions to double bonds to initiate a radical oxidation chain reaction [25]. This leads to the pollutant transformation into carbon dioxide, water, inorganic ions, or harmless and biodegradable byproducts, such as carboxylic acids [22].

(1) $$M+H_{2}O\;\to \;M{[}^{•}\mathrm{OH}]+H^{+}+e^{-}$$

The radical availability will depend on the anodic overvoltage (*η*_an_), which is the difference between the oxidation potential (*E*_an_) and its equilibrium value (*E*_eq_) that varies in function of the material. Anodes are categorized into active or nonactive, based on the *E*_*an*_ needed to oxidize H_2_O to O_2_ [26, 27]. When *E*_an_ < 2 V versus SHE, which corresponds to a low anodic overpotential, the anode is considered active, and ^•^OH will be chemically adsorbed. This involves strong interactions with the electrode, preventing ^•^OH desorption and making it unavailable for pollutant degradation, as described by Equation (2). Platinum‐, iridium oxide‐, and ruthenium‐based electrodes are considered active anodes [24].

(2) $$M{[}^{•}\mathrm{OH}]+H_{2}O\;\to \;M+O_{2}+\;3H^{+}+3e^{-}$$

When *E*_an_ ≥ 2 V versus SHE, which corresponds to a high anodic overpotential, the anode is considered nonactive, and ^•^OH are physically adsorbed on the electrode surface. The weak interactions increase ^•^OH non‐sorption, making them available to react with the compounds at the electrode surface, as described by Equation (3), where R′ is an oxidation product of R. Boron‐doped diamond, sub‐stoichiometric titanium dioxide (Ti_*n*_O_2n−1_, with *n* = 4 or 5), known as Magnéli phase, and lead dioxide (PbO_2_) are nonactive anodes.

(3) $$M{[}^{•}\mathrm{OH}]+R\;\to \;M+\mathrm{R\prime}$$

**FIGURE 2:**
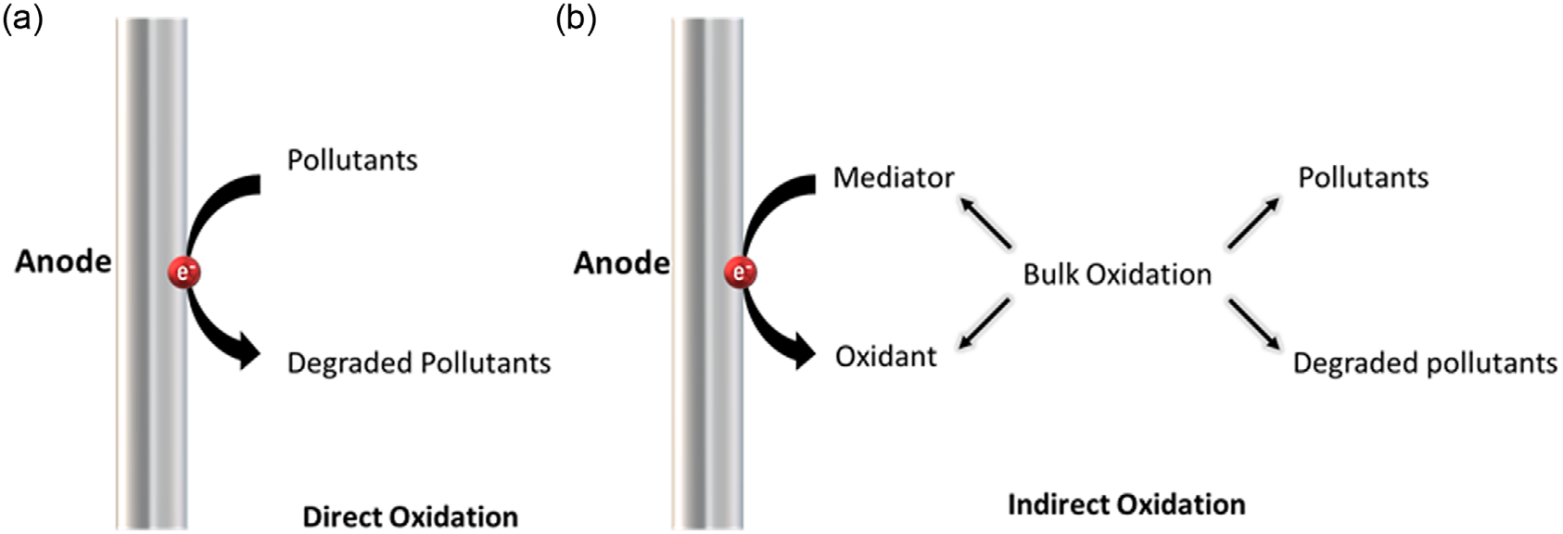
Direct (a) and indirect (b) oxidation. In the direct process, electrons are transferred to the pollutants adsorbed on the anode. In the indirect process, electron transfer occurs through electroactive species, such as Cl^•^ and SO_4_^•−^, produced by the electrolysis of Cl^−^ and SO_4_^2−^. The efficiency of both mechanisms is influenced by the electrode material, electrolyte solution composition, current density, and pollutant type. Adapted with permission [28]. Copyright 2009, John Wiley and Sons.

Considering only the anode side, degradation can occur via direct or indirect oxidation (Figure 2). Depending on the electrolysis duration, the effluent toxicity can be enhanced by the formation of byproducts that must be removed, for instance, through a separation step [28].

In direct oxidation, electrons are directly transferred to the pollutants adsorbed onto the anode [18]. However, the pollutant needs to be electroactive at the anode when the potential is applied, which is not the case for most water pollutants [26]. Oxidation can also occurs via ^•^OH produced on the anode surface, i.e., mediated oxidation. In indirect oxidation, the electron exchange occurs via electroactive species, such as Cl^•^, SO_4_^•^^−^ that can be generated by electrooxidation of Cl^−^ and SO_4_^2*−*^. Catalytic metal mediators, such as silver and iron, can also be used to generate ^•^OH [29].

**FIGURE 3:**
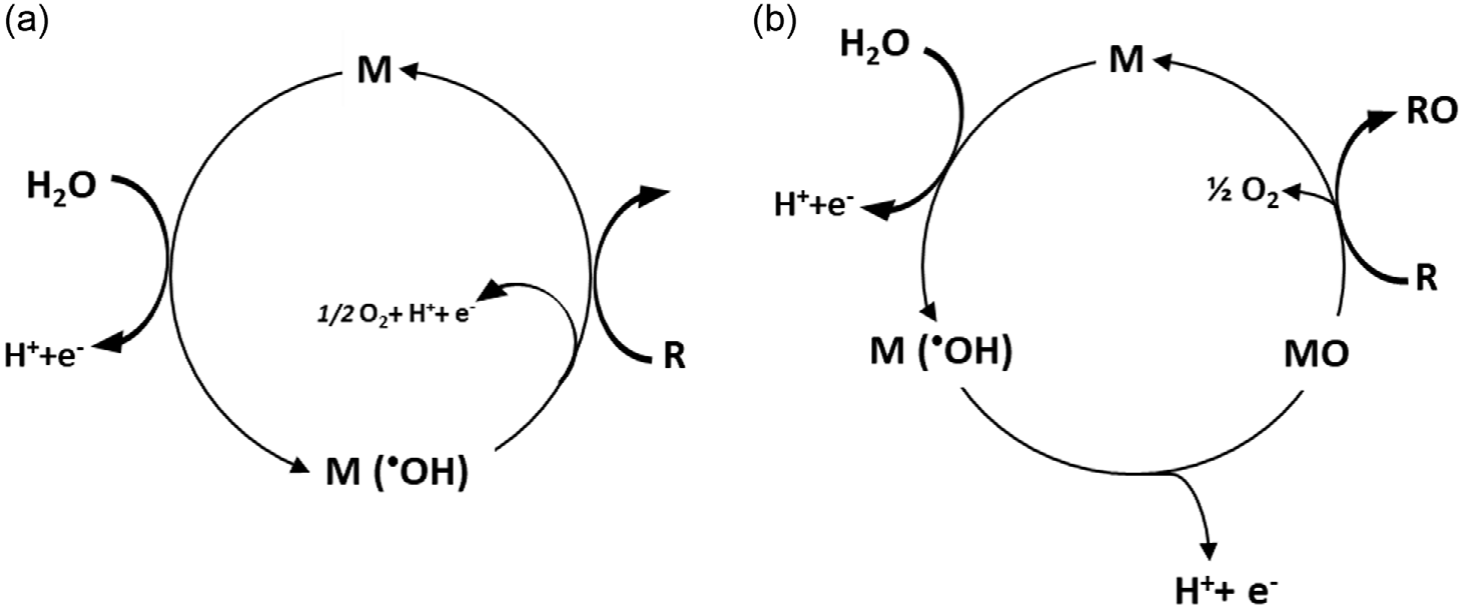
Pollutant degradation by anodic electrooxidation using active (a) and nonactive anodes (b). In the presence of an active anode, the physically adsorbed hydroxyl radicals (^•^OH) completely degrade the oxidizable compounds present in the solution. Conversely, in the presence of a nonactive anode, chemically sorbed ^•^OH lead to the formation of metal oxide groups at the electrode surface and of protons as a selective oxidation of pollutants. Note that in the absence of organic oxidation, dioxygen is generated. Adapted with permission [27]. Copyright 1994, Elsevier. *M(^•^OH): metal hydroxide, M: metal, MO: metal oxide, RO: organic radical, R: radical.

The electrooxidation mechanisms of active and nonactive anodes were first described in 1994 (Figure 3) [26, 27]. Phenolic compounds were among the first pollutants used to investigate their degradation mechanism by electrochemical advanced oxidation processes as mentioned in the review article by Martinez‐Huitle and Ferro [22].

**FIGURE 4:**
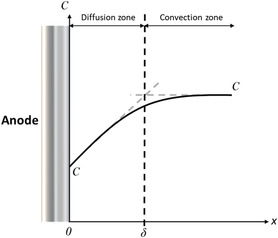
Mass transport zone. The reaction rate is influenced by the pollutant movement toward the electrode and the electron exchange occurring at the electrode surface. In the bulk solution, pollutants are transported by convection, facilitating their approach to the electrode for oxidation. However, their concentration near the anode decreases because of degradation, and diffusion becomes the primary mechanism for their movement to the electrode surface.

The electrooxidation kinetics is governed by the pollutant transfer to the interface by convection, diffusion or migration, and by the interfacial electron exchange [26, 30]. Pollutant transfer by convection requires a mechanical force (e.g., stirring). Movement by diffusion is based on the chemical potential difference, and movement by migration only concerns ionic species governed by an electric field [26]. As the pollutant content is low compared with the total ionic charge, its movement by migration is negligible. In bulk solutions, pollutants have the same concentration, and mass transport is only by convection. When approaching the anode, the pollutant concentration tends to decrease because of degradation, inducing mass transport by diffusion (Figure 4).

The efficiency of electrooxidation can be limited by the pollutant diffusion at the electrode, especially in the case of micropollutants (i.e., at very low concentration). To overcome this issue, a preconcentration step, for instance by adsorption, can be carried out using activated carbon. Alternatively, porous electrodes in a flow‐through configuration, also known as reactive electrochemical membranes, can be used [30]. Compared with the traditional flow‐by configuration, the electrochemical membrane efficiency is mainly explained by the high mass transfer rate and the porous electromaterial with high electroactive surface area [31].

### 2.1 Influence of Operating Conditions on Anodic Oxidation

The electrooxidation of a large variety of pollutants (Table 2) has been studied in different conditions (pH, temperature, current density and electrolyte type) and with different anode types to identify the parameters that influence the process efficiency. For instance, Sopaj et al. evaluated different electrodes (carbon‐felt, carbon fiber, carbon graphite, platinum, lead dioxide, dimensionally stable Ti/RuO_2_–IrO_2_ anode, and boron‐doped diamond) for amoxicillin mineralization [45]. They found that boron‐doped diamond was the best anode, generating a large amount of ^•^OH and other oxidants, whereas the dimensionally stable Ti/RuO_2_–IrO_2_ anode was the least efficient. Indeed, only 50% of amoxicillin was oxidized in the best operating conditions by the Ti/RuO_2_–IrO_2_ anode. The Magnéli phase (sub‐stoichiometric titanium dioxide) also was an efficient anode, generating a large quantity of physisorbed ^•^OH, and was less expensive than boron‐doped diamond [44].

**TABLE 2:** Removal efficiency of different anode materials for various pollutants in electrooxidation process. Several pollutants, such as indigo carmine, 4‐chlorophenol, quinoline, tetracycline, and iohexol, are completely removed using specific anodes. This highlights the effectiveness of electrooxidation and the importance of choosing suitable anodes for optimal pollutant degradation.

Pollutant	Anode	Removal efficiency	Reference
Indigo carmine	Ti/IrO_2_–SnO_2_–Sb_2_O_5_	100%	[32]
Bisphenol A	Boron‐doped diamond	90%	[33]
Methyl orange	Exfoliated graphite	98.6%	[34]
Methylparathion	Ti/Pt	82%	[35]
Para‐chlorobenzoic acid	Boron‐doped diamond	98.4% of COD	[36]
4‐Chlorophenol	Boron‐doped diamond	100%	[37]
	Lead dioxide	100%
Aniline	Boron‐doped diamond	80%	[38]
Lignosulfonate	Boron‐doped diamond	80% of TOC	[39]
Quinoline	Codoped PbO_2_	100 %	[40]
Tetracycline	Ti/IrO_2_	∼99%	[41]
Iohexol	Boron‐doped diamond	100%	[42]
Acid blue 22	Boron‐doped diamond	97% of COD	[43]
Tetracycline	Ti_4_O_7_	100%	[44]

Abbreviations: COD: chemical oxygen demand, TOC: total organic carbon.

Electrooxidation is promoted by acidic conditions. The redox potential of ^•^OH/H_2_O can be expressed as a function of the pH according to the Nernst relationship (Equation (4))

(4) $$E^{0}{(}^{•}{\mathrm{OH}}_{\mathrm{aq}}\;/H_{2}O)=2.59-0.059\text{pH}$$

Thus, at lower pH, the value of $E^{0}{(}^{•}{\mathrm{OH}}_{\mathrm{aq}}/H_{2}O)$ will induce higher oxidative levels. Moreover, higher temperatures improve the pollutant degradation [46], as described by Equation (5), the Arrhenius equation: *k* is the rate constant, *T* is the temperature, A is a constant, *E*_a_ is the activation energy, and R is the universal gas constant [47].

(5) $$\mathrm{lnk}=-\frac{E_{a}}{RT}+\mathrm{lnA}$$

The supporting electrolyte contributes to increase the solution conductivity, and can also improve removal efficiency by generating oxidizing species that react with the pollutant in water. For instance, active chlorine species can be generated from chloride ions [48]. However, using chloride as electrolyte results in the production of organochlorine compounds that are persistent and toxic for the environment.

The current density, defined as the electric current that flows through a unit area of the anode, influences the quantity of the oxidative species produced [18]. On the other hand, Pierpaoli et al. showed that the rate of pollutant removal improves as the current density increases [18].

### 2.2 Anodic oxidation of Recalcitrant Pollutants: The Case of PFAS

PFAS are widely used in many industrial applications. They are amphiphilic with a hydrophilic functional head and a hydrophobic tail that contains fluorine atoms. They are highly stable and present major risk to human and environment. Furthermore, they resist to conventional water treatment techniques due to the strong bond between carbon and fluorine (485 kJ/mol) [49]. Conversely, they can be efficiently degraded by electrooxidation (up to 99%) [18].

Perfluorooctanoic acid and perfluorooctanesulfonic acid are among the PFAS most frequently detected in the environment [18]. Equations (6)–(15) and (16)–(28) describe the degradation mechanisms of perfluorooctanoic acid and perfluorooctanesulfonic acid, respectively [50]. During perfluorooctanoic acid degradation, perfluorooctanoic acid radicals are first formed by direct electron transfer from the anode (Equation (7)), followed by Kolbe decarboxylation (Equation (8)). Then, perfluoro radicals react with oxygen (Equations (9)–(12)) or other radicals (Equations (13)–(15)), inducing an unzipping CF_2_ cycle until complete mineralization. Perfluorooctanesulfonic acid degradation also includes hydrolysis, hydroxylation, radical reaction, and Kolbe decarboxylation. Veciana et al. proposed that functional groups, such as carboxylate against sulfonate, can impact PFAS behavior through electrochemical oxidation [18]. Conversely, Liang et al. hypothesized that perfluorooctanesulfonic acid has more affinity to be adsorbed on the anode, contributing to its rapid degradation compared with perfluorooctanoic acid. Perfluorooctanesulfonic acid degradation involves hydrolysis, hydroxylation, desulfonation steps, in addition to direct electron transfer and Kolbe‐type reaction (Equations (16)–(28)), leading to the formation of perfluoroalkyl radicals similar to those observed for perfluorooctanoic acid. The cleavage of the C—S bond is a key step, after which the degradation pathway converges toward the progressive defluorination and shortening of the carbon chain. However, when the effluent contains both perfluorooctanoic acid and perfluorooctanesulfonic acid, perfluorooctanoic acid degradation rate is higher, likely due to the higher ^•^OH affinity for these substances [51].

The pollutant degradation can be influenced by the PFAS functional head group and by the hydrophobic tail length. Wang et al. investigated the degradation of perfluorobutanoic acid, perfluoropentanoic acid, perfluorohexanoic acid, perfluoroheptanoic acid and perfluorooctanoic acid [52] that have the same functional group, but different carbon chain lengths. They found that compounds with a longer carbon chain have better reactivity, explaining the recalcitrant behavior of PFAS with shorter chains [53].

(6) $${\mathrm{Ti}}_{4}O_{7}+H_{2}O\;\;\to \;\;{\mathrm{Ti}}_{4}O_{7}{(}^{•}\mathrm{OH})+H^{+}+e^{-}$$

(7) $$C_{7}F_{15}{\mathrm{COO}}^{-}\;\;\to \;\;C_{7}F_{15}{\mathrm{COO}}^{•}+e^{-}$$

(8) $$C_{7}F_{15}{\mathrm{COO}}^{•}\;\;\to \;\;C_{7}F_{15}^{•}+{\mathrm{CO}}_{2}$$

(9) $$C_{7}F_{15}^{•}+O_{2}\;\to \;\;C_{7}F_{15}{\mathrm{OO}}^{•}$$

(10) $$C_{7}F_{15}{\mathrm{OO}}^{•}+R_{F}{\mathrm{COO}}^{•}\;\to \;\;C_{7}F_{15}O^{•}+R_{F}{\mathrm{CO}}^{•}+O_{2}$$

(11) $$C_{7}F_{15}O^{•}\;\to \;\;C_{6}F_{13}^{•}+{\mathrm{COF}}_{2}$$

(12) $${\mathrm{COF}}_{2}+H_{2}O\;\to \;{\mathrm{CO}}_{2}+2\mathrm{HF}$$

(13)

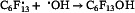

(14) $$C_{6}F_{13}\mathrm{OH}\;\to \;\;C_{6}F_{11}O+\mathrm{HF}$$

(15) $$C_{6}F_{11}O+H_{2}O\;\to \;\;C_{5}F_{11}{\mathrm{COO}}^{-}+\mathrm{HF}+H^{+}$$

(16) $$C_{8}F_{17}{\mathrm{SO}}_{3}^{-}+H^{•}\;\to \;C_{8}{\mathrm{HF}}_{16}{\mathrm{SO}}_{3}^{-}+F^{-}+e^{-}$$

(17)

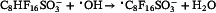






(18)

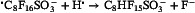






(19)

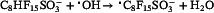






(20)

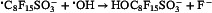

(21) $${\mathrm{HOC}}_{8}F_{15}{\mathrm{SO}}_{3}^{-}+2F^{-}\;\to \;{\mathrm{FOC}}_{8}F_{15}{\mathrm{SO}}_{3}^{-}+{\mathrm{HF}}^{-}$$

(22)

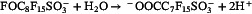






(23)

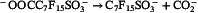






(24)

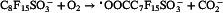






(25)

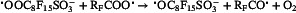






(26)

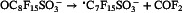

(27) $${\mathrm{COF}}_{2}+H_{2}O\;\to \;2\mathrm{HF}+{\mathrm{CO}}_{2}$$

(28) $$C_{7}F_{15}{\mathrm{SO}}_{3}^{-}+\;H_{2}O\;\to \;C_{7}F_{15}^{•}+{\mathrm{SO}}_{4}^{2-}+2H^{+}$$

Moreover, studies using laboratory‐prepared effluents containing PFAS showed that the fluoride concentration at the electrooxidation end was lower than expected. This difference was explained by the presence of undetectable degradation byproducts or by the conversion of F^−^ to volatile F_2_ or HF. Alternatively, PFAS can be adsorbed on the walls of electrolytic cells [18, 53].

Most of the studies on PFAS degradation by electrooxidation were performed using synthetic effluents; however, real water matrixes are more complex. The presence of competing pollutants can affect the degradation efficiency, thus requiring the optimization of the operating conditions. For instance, Pierpaolo et al. reported that the reaction time or energy density must be increased because inorganic salts and organic matter can interfere with the electrooxidation process [50]. They showed that in landfill leachates treated by electrochemical oxidation on boron‐doped diamond electrodes, PFAS removal efficiency increased from ∼50% at 25 mA/cm^2^ to ∼78% for perfluorooctane sulfonic acid and 80% for perfluorooctanoic acid when the current density was raised to 75 mA/cm^2^. Schaefer et al. investigated the degradation of these two pollutants in natural groundwater contaminated by aqueous film‐forming foams used for fire training exercises [53]. They also found that their degradation rate increased when higher current densities were applied. Moreover, when using a boron‐doped diamond anode, perchlorates were generated during the electrooxidation process due to the presence of chloride ions in the water source. However, their concentration could be reduced using sand columns, up to 99.9% after 9 day of treatment [54].

PFAS concentration also depends on the location and nature of the water source: ng/L in groundwater and surface water and µg/L in water bodies close to industrial sites [55]. Therefore, a preconcentration step may be required for a more efficient electrooxidation process and to reduce the energy needed, especially when using dense plate electrodes where the efficiency is limited by the mass transfer. In this type of reactor configuration, even the plate distance can influence the energy consumption and degradation efficiency. Lin et al. showed that by shortening the distance between plates, the energy consumption is decreased and the degradation rate increased [56].

Energy consumption is a key factor, particularly for scaling up the electrooxidation process as well as the presence of natural organic matter, inorganic ions and other pollutants, water pH, and PFAS concentration in the effluent.

These results highlight that the composition of real water plays a critical role in electrooxidation performance. Particularly, some ions, such as SO_4_^2^^−^ and HPO_4_^2^^−^, can act as scavengers of hydroxyl radicals, reducing their availability for PFAS degradation. In addition, natural organic matter competes with PFAS for reactive species and electrode active sites, leading to lower selectivity and increased energy consumption. Mass transfer limitations are also more pronounced in real water samples, where PFAS are typically present at very low concentrations (ng L^−1^ to µg L^−1^), further reducing treatment efficiency. Altogether, these factors demonstrate that PFAS electrooxidation in real wastewater requires higher energy input and careful control of operating parameters, and often benefits from the integration with complementary treatment processes to achieve effective and safe pollutant removal.

## 3 Electro‐Fenton Process

The Fenton process is an advanced oxidation process where ^•^OH are generated using the Fenton's reagent (H_2_O_2_ + Fe^2+^), as described in Equation (29) [57]

(29) $$H_{2}O_{2}+{\text{Fe}}^{2+}\;\to \;{\text{Fe}}^{3+}+{\text{OH}}^{\text{–}}{+}^{•}\text{OH}$$

This process is an effective system for generating ^•^OH to destroy persistent/toxic micropollutants because the radical formed is a nonselective strong oxidant (*E*° = 2.80 V/SHE) that can react with any organic compound leading to its mineralization [26]. However, it has several drawbacks, such as the need of large amounts of chemicals, generation of high quantities of sludge, high risks related to reagent transport and storage (mainly H_2_O_2_), and consumption of the formed ^•^OH and Fenton's reagent in wasting reactions (Equations (30) and (31)) [57]



(30)

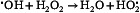






(31)

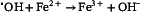




To overcome these drawbacks and improve process efficiency, a new Fenton‐related process called “electro‐Fenton,” was developed by combining the Fenton process and electrochemistry for the in situ generation of H_2_O_2_ (Equation (32)) [58, 59]

(32) $$O_{2}+2H^{+}+2e^{\text{–}}\;\to \;H_{2}O_{2}$$

Additionally, the Fenton reaction is catalyzed through Fe^2+^ regeneration via electroreduction of the Fe^3+^ generated in the Fenton reaction following Equation (33) [58, 59]

(33) $${\text{Fe}}^{3+}+e^{\text{–}}\;\to \;{\text{Fe}}^{2+}$$

The electro‐Fenton process was developed by Brillas’ and Oturan's teams in its gas diffusion cathode and carbon felt cathode versions, respectively, in the early 2000s [58, 60]. It is frequently used to remove persistent organic pollutants from wastewater [19, 25]. Besides minimizing the wasting reactions (Equations (30) and (31)) and in‐situ generating reagents, this process displays high oxidation efficacy and outstanding mineralization capacity [61].

**FIGURE 5:**
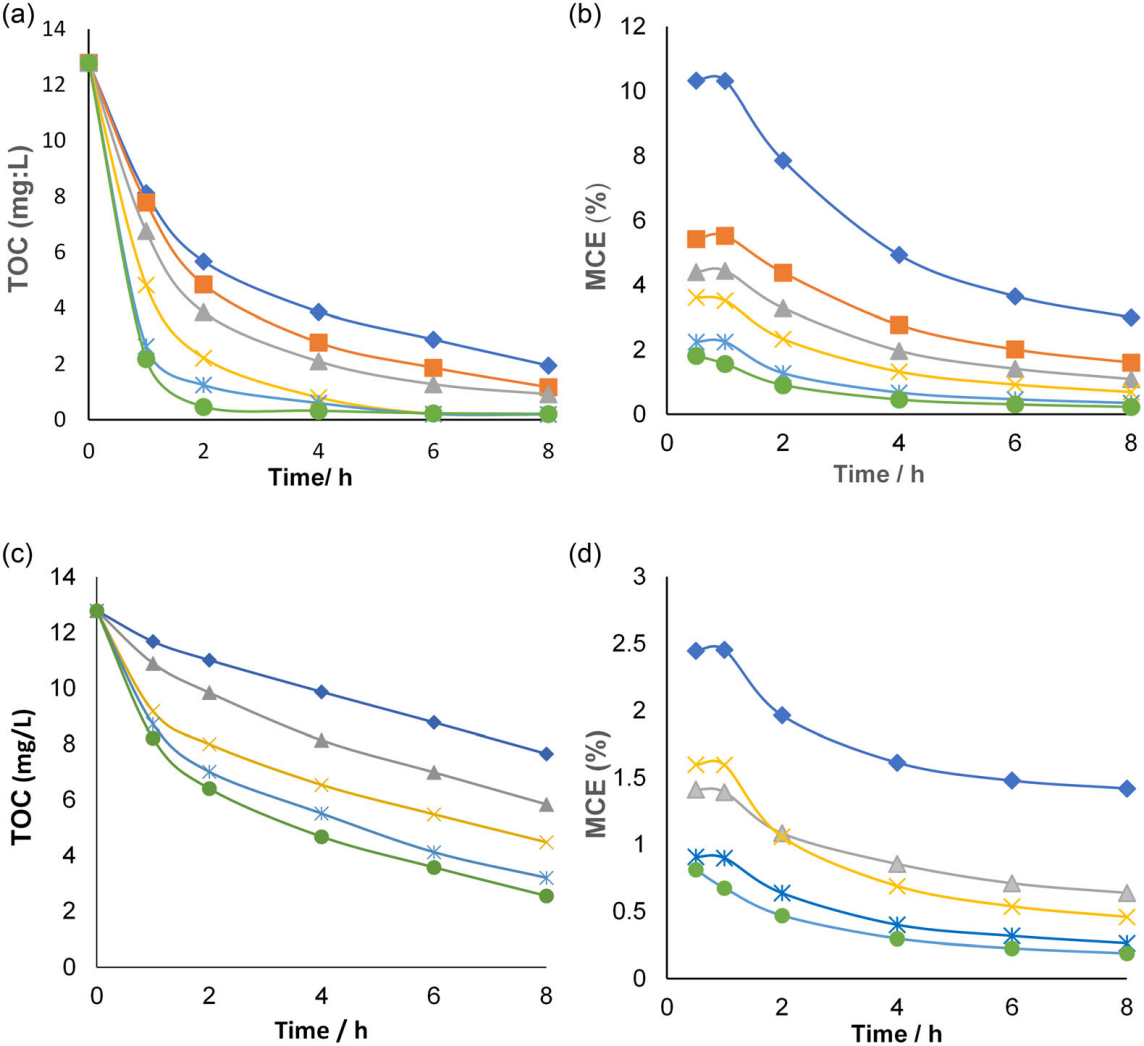
Effect of current and anode material on mineralization efficiency during the electro‐Fenton treatment of 0.1 mM fluometuron (a pesticide) using boron‐doped diamond and Pt anodes. Evolution of mineralization rate and mineralization current efficiency (%) with boron‐doped diamond (a,b) and Pt (c,d) anodes, respectively. Experimental conditions: pH 3; [Fe^2+^] = 0.1 mM; [Na_2_SO_4_] = 50 mM; V = 230 mL; *I* (mA): 100 (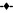); 200 (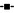); 300 (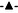); 500 (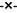); 1000 (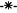); 1500 (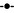). The electro‐Fenton process provides significantly better mineralization power when using a boron‐doped diamond anode compared with a Pt anode. The mineralization rate increases with the current and the mineralization current efficiency decreases at high currents, leading to high‐energy consumption. Reproduced with permission [63]. Copyright 2017, Elsevier. TOC: total organic carbon, MCE: mineralization current efficiency.

The electro‐Fenton process efficiency is influenced by several parameters. Some of these parameters, such as the solution pH (≈3), the catalyst nature and concentration (Fe^2+^ at 0.1−0.2 mM), and the electrolyte (0.05 M Na_2_SO_4_), have already been well established [59, 60, 61, 62]. In addition, the nature of the electrodes is also important. Carbonaceous cathodes, such as carbon felt, carbon‐polytetrafluoroethylene, carbon fiber, graphite, and reticulated vitreous carbon, promote H_2_O_2_ generation [62]. Similarly, anode materials with high overpotential for O_2_ evolution reactions, for example, boron‐doped diamond, can effectively produce heterogeneous hydroxyl radicals M(^•^OH), according to Equation (1), in addition to homogeneous ^•^OH generated in solution. Boron‐doped diamond anodes significantly increase the mineralization capacity of the process (Figure 5).

The applied current also strongly affects the process efficacy (Figure 5) because it governs the generation rate of homogeneous (Equations (32) and (33)) and heterogeneous ^•^OH (Equation (34)) and consequently the formation rate of homogeneous and heterogeneous ^•^OH. Higher currents increase the mineralization rate, monitored by quantifying the total organic carbon (TOC) in the solution, but decrease the mineralization current efficiency, thus requiring more energy. Therefore, mineralization rate and current efficiency must be balanced to improve the system cost‐effectiveness.
(34)

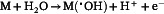




Since the first applications in the early 2000s, the electro‐Fenton process has been continuously developed and applied successfully for the removal of many pollutant types, from pesticides to personal care products [64]. Several reviews have been published on different advances related to the development of the electro‐Fenton process [65, 66, 67, 68, 69]. The objective here is to provide a concise overview of various research directions to improve the applicability of the electro‐Fenton process for water treatment in viable operating conditions.

### 3.1 Heterogeneous Electro‐Fenton Process

A dissolved iron source (Fe^2+^/Fe^3+^) is used in the electro‐Fenton process; however, iron solubility is poor at near‐neutral pH values. Therefore, the process must be carried out at acidic pH (optimal value close to 3.0), and a pH adjustment step is usually required before the electro‐Fenton‐based treatment of real effluents. This can modify the solution composition. An alternative is the heterogeneous electro‐Fenton process in which a solid iron source is used as catalyst to promote the Fenton reaction through surface reaction at near‐neutral pH (Equation (35)) [17]

(35) $$\equiv {\text{Fe}}^{\text{II}}-\text{OH}+H_{2}O_{2}\;\to \equiv {\text{Fe}}^{\text{III}}-\text{OH}{+}^{•}\text{OH}+{\text{OH}}^{-}$$

Different approaches have been tested to identify the best solid iron source. A typical approach is the use of natural iron‐containing minerals, such as pyrite (FeS_2_) or chalcopyrite (CuFeS_2_) [70, 71]. These minerals provide the iron source and decrease the pH to about 3 (Equation (36)). Then, the process is mainly driven by a homogeneous reaction in the solution with the iron released from the minerals. These minerals constitute a natural and low‐cost iron source that may avoid the initial pH adjustment step of the effluent, depending on the buffer capacity of the effluent. However, a pH adjustment step will be required after the treatment for the effluent safe discharge at near‐neutral pH.

(36) $$2{\text{FeS}}_{2}\;+\;7O_{2}\;+\;2H_{2}O\;\;\to \;\;2{\text{Fe}}^{2+}\;+\;4{\text{SO}}_{4}^{2\text{–}}\;+\;4H^{+}$$

Other solid iron sources have been tested, including natural minerals (e.g., goethite and other iron oxides), synthetic nanoparticles (to optimize the specific surface area available for surface reactions), and iron‐supported catalysts. Their effectiveness depends on reactions at the catalyst surface and the homogeneous reaction in the bulk. Reactions at the catalyst surface become predominant at near‐neutral pH [17, 61, 72, 73, 74].

**FIGURE 6:**
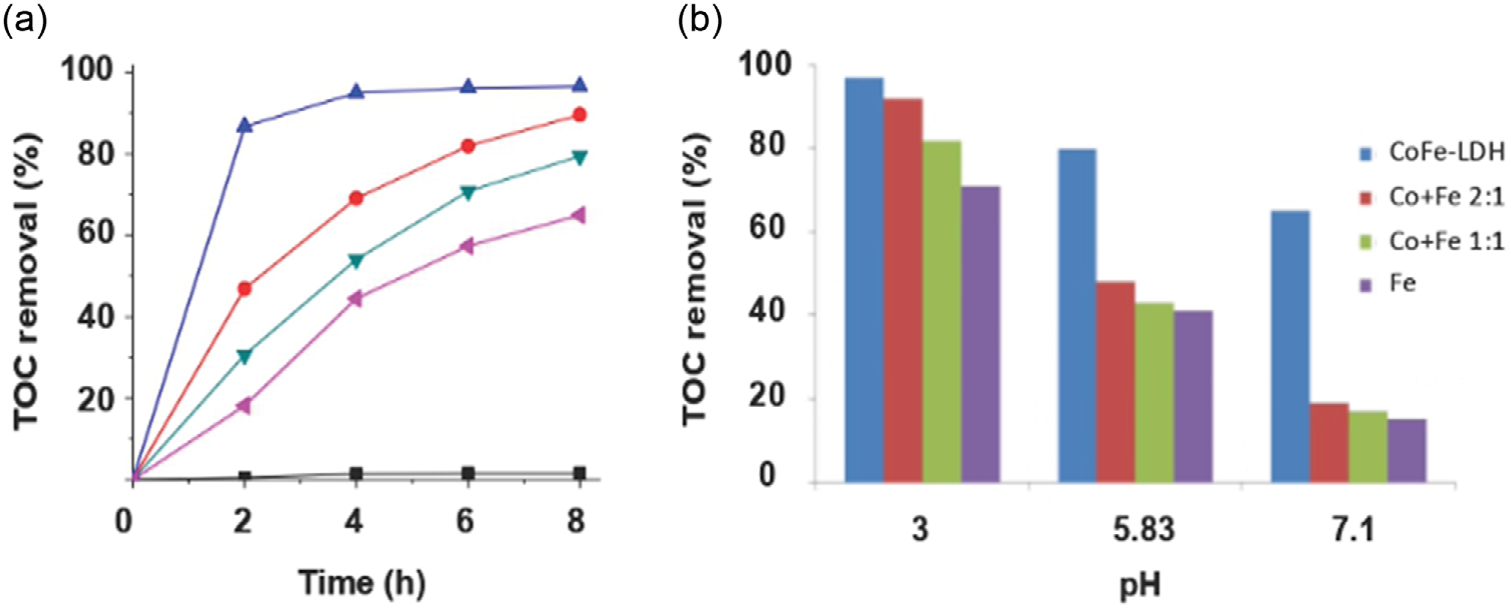
(a) Effect of the pH on Acid Orange 7 mineralization in the presence of a cobalt‐iron‐ layered double hydroxide (LDH) grown on carbon felt (CoFe‐LDH) (25:12.5 Co/Fe initial molar concentration) at 90°C, for 7 h: 0 mA (black), pH 2 (red), pH 3 (blue), pH 5.83 (green), and pH 7.1 (purple) and (b) total organic carbon removal (%) at different pH values using a heterogeneous electro‐Fenton process with the CoFe–LDH cathode (blue) and a homogeneous electro‐Fenton process with the raw carbon felt cathode and Fe^2+^/Co^2+^, as catalysts. Adapted with permission [75]. Copyright 2017, Royal society of chemistry. TOC: total organic carbon, h: hour.

Another approach is the impregnation of the iron source directly onto the cathode materials that can generate H_2_O_2,_ mainly carbonaceous materials. These materials usually display great electrocatalytic features. For example, a cobalt–iron‐layered double hydroxide grown on a carbon cathode [75] strongly increased the mineralization of Acid Orange 7 compared with the homogeneous electro‐Fenton process, particularly at near‐neutral pH (Figure 6). Other material types also have been developed, including Fe‐containing particle electrodes and other modified carbonaceous materials [76, 77]. However, as these electrode materials must be used for water treatment applications, their long‐term stability is still a major issue.

### 3.2 Activated Carbon Regeneration

Two drawbacks of advanced oxidation processes, including the electro‐Fenton process, are related to (i) the formation of toxic halogenated compounds (e.g., ClO_3_^−^, ClO_4_^−^ and organohalogen compounds) and (ii) the low energy efficiency when treating pollutants at low concentration because of mass transport limitations, particularly in anodic oxidation [65]. These problems could be limited by exploiting the electro‐Fenton process for activated carbon regeneration. In this case, the target effluent is continuously treated through adsorption on activated carbon that allows removing organic compounds with low energy/chemical consumption. Then, the spent activated carbon is used as cathode for punctual regeneration through the electro‐Fenton process in order to be reused in other adsorption cycles. This strategy presents several advantages, including: (i) lower consumption of activated carbon that may reduce the carbon footprint of the whole process; (ii) degradation and mineralization of the target pollutants that are initially only separated from the effluent during the adsorption step; (iii) higher energy efficiency of the electro‐Fenton process due to the preconcentration step during the adsorption that increases the pollutant concentration; and (iv) possibility to select the most appropriate inorganic matrix for the regeneration solution (e.g., without chloride salts) in order to avoid the formation of undesired byproducts during the electro‐Fenton process.

**FIGURE 7:**
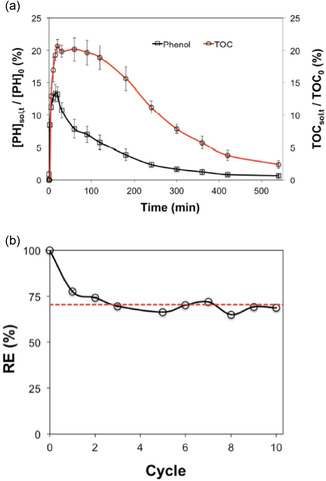
(a) Changes in phenol and total organic carbon concentrations in the regeneration solution ([PH]_sol,t_ and TOC_solt,t_) at different time points during the electro‐Fenton process‐mediated regeneration of spent activated carbon fibers. [PH]_0_ and TOC_0_ are the initial concentrations (including the adsorbed compounds). (b) Regeneration efficiency (i.e., the adsorption capacity of the regenerated activated carbon relative to the initial adsorption capacity) in function of the number of adsorption/regeneration cycles. Adapted with permission [79]. Copyright 2018, American Chemical Society. PH: phenol, TOC: total organic carbon.

The feasibility of activated carbon regeneration has been demonstrated using granular activated carbon or activated carbon fibers [78, 79, 80, 81]. For example, Figures 7a,b show the concentration changes of phenol and TOC in the solution during activated carbon regeneration and the regeneration effectiveness based on the activated carbon adsorption capacity measurement. These effects are due to (i) the electrochemically‐enhanced pollutant desorption through electrostatic interactions; (ii) the pollutant oxidation in the bulk and continuous shift of the sorption equilibrium; (iii) the sorbed pollutant oxidation and formation of more hydrophilic byproducts; and (iv) the low oxidation/alteration of the activated carbon surface owing to the material cathodic polarization [80, 82]. Overall, the data obtained from laboratory‐scale experiments highlight the potential of such treatment strategy. However, more studies are required to: (i) choose/develop materials with suitable characteristics for adsorption and electrochemical regeneration, (ii) understand the reaction and sorption phenomena in the activated carbon bed, (iii) design and validate reactors for full‐scale applications, and (iv) validate the relevance of such approach with real effluents.

It is important to note that this approach faces scalability challenges. Indeed, the reactor characteristics (e.g., diameter and height of the activated carbon bed) must be appropriately designed for both the adsorption phase and the regeneration phase, during which issues, such as current distribution and electrical interconnection throughout the material, may arise. The positioning of the counter‐electrodes also must be optimized, and fluid circulation must take into account the removal of gas bubbles generated at the electrode surfaces. Furthermore, treatment objectives and operating conditions must be clearly defined to ensure the process viability, particularly in terms of energy consumption. For instance, the complete mineralization of organic pollutants in the regeneration solution may be too energy‐intensive. The results by Trellu et al. at the bench scale indicated that the removal of organic compounds adsorbed on activated carbon fibers can be achieved with a mineralization current efficiency of 35% and an energy consumption of 200 kWh/kg of TOC mineralized. In this context, the evolution of the regeneration solution biodegradability could become an important parameter to reduce energy consumption, as discussed in the next section. Lastly, the lifetime of the filter (i.e., the number of possible regeneration cycles) will also be a key factor in assessing the overall process viability. Some studies indicated that at the bench scale, the adsorption cycle could be maintained from a few to 10 cycles [79]. However, no study has really explored the limits of the system.

To date, evaluations of this process type at the pilot scale have not been reported.

### 3.3 Combining the Electro‐Fenton Process With a Biological Process

In the last decade, the electro‐Fenton process has been combined with biological treatments as a potential strategy for improving cost‐effectiveness, particularly for treating industrial effluents rich in recalcitrant pollutants. In this strategy, pollutants are converted by the electro‐Fenton process into more biodegradable byproducts, which are then destroyed using a biological treatment (e.g., activated sludge). The electro‐Fenton process usually leads to much faster degradation kinetics compared with the mineralization kinetics [83]. Moreover, its final byproducts, mainly short‐chain carboxylic acids, react slowly with ^•^OH, but are easily removed by a biological treatment [60, 84]. Therefore, this strategy allows reducing the electric charge and the energy consumed during the electro‐Fenton step. This approach was tested for the treatment of pharmaceutical compounds/effluents, stabilized landfill leachates, ionic liquids, and other synthetic or real effluent solutions [85, 86, 87, 88]. Research is now focused on developing continuous pilot‐scale reactors and analytical tools to optimize this two‐step strategy. The use of heterogeneous catalysts or cathodes modified by solid iron‐based catalysts for the heterogeneous electro‐Fenton process and its coupling with other processes, for example biological processes, will overcome the limitations of the electro‐Fenton process.

## 4 Photo‐Electrooxidation

Photo‐electrooxidation, also known as photo‐electrocatalysis or photo‐electrochemical oxidation, relies on the generation of reactive species, such as ^•^OH, superoxide anions, photogenerated holes, and electrons at the anode surface, that play a crucial role in breaking down complex emerging pollutants into less harmful or mineralized products. Its capacity to produce strong reactive species explains its effectiveness and suitability for addressing water pollution issues [89, 90].

**FIGURE 8:**
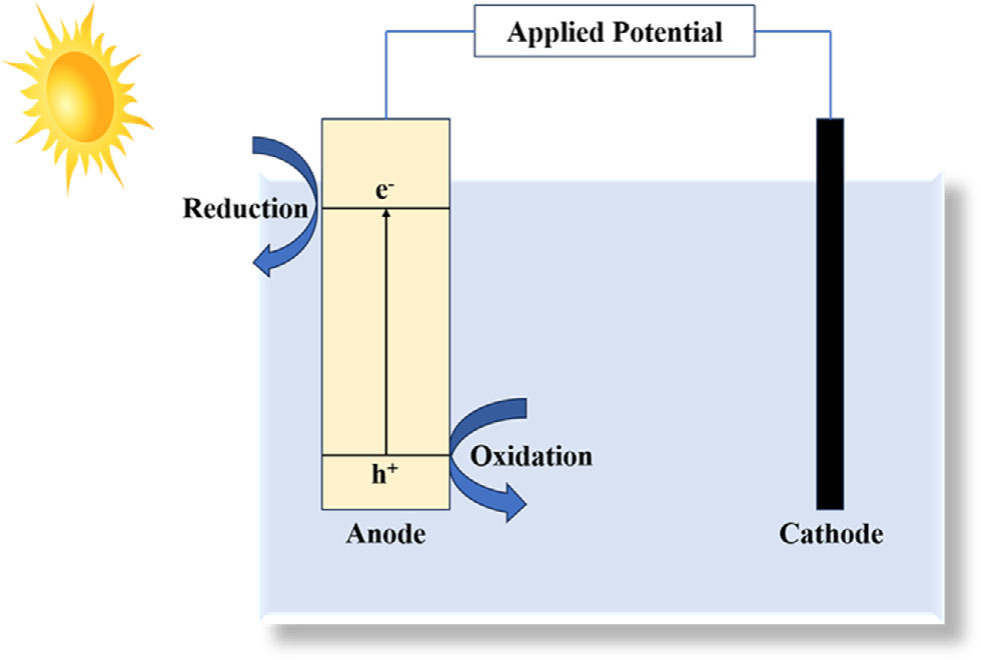
Photoelectrochemical oxidation system. The photoelectrode, often made of semiconducting materials, absorbs light and generates electron–hole pairs. These charge carriers facilitate oxidation reactions at the anode where pollutants are degraded. The cathode typically supports reduction reactions to balance the system. The electrolyte solution conducts ions between the electrodes, completing the circuit and enhancing the overall efficiency of the oxidation process. Adapted with permission [91]. Copyright 2017, Elsevier. e^−^: electrons, h^+^: holes.

Unlike the electro‐Fenton process, a predominantly cathodic process, photo‐electrooxidation is an anodic process that combines photocatalysis and electrochemical oxidation at the photoanode (Figure 8) to concomitantly improve the oxidation efficiency and pollutant degradation. Compared with other advanced oxidation processes, photo‐electrooxidation can degrade a wide range of organic pollutants in water and its energy consumption is usually lower compared to anodic oxidation. Moreover, the photocatalyst can be easily recovered [91]. Photo‐electrooxidation has been tested for removing pharmaceuticals (e.g., antibiotics and analgesics), dyes, and pesticides [92, 93, 94, 95, 96, 97].

The photoanode includes a photocatalyst that is usually coated or electrodeposited on a conducting substrate, such as fluorine‐doped tin oxide and indium tin oxide. The semiconductor material used in the photoanode is electroactive and absorbs light energy that is greater than its bandgap energy.

For oxidation, the semiconductor absorbs light energy. This allows electrons to move to the conduction band, leaving a positively charged vacancy (i.e., holes) in the valence band. These holes (h^+^) and electrons (e^−^) can directly degrade pollutants or react with water molecules and absorbed oxygen to facilitate the pollutant removal from water [98], as shown in Equations (37)–(42) [89, 99]. The combined photo‐electrochemical oxidation process helps to introduce a bias potential that limits the fast recombination rate of electrons and holes, a major problem that can affect the semiconductor degradation efficiency [100]. In conclusion, the combination of photocatalysis and electrochemical oxidation ensures the efficient generation and use of reactive species, making of photo‐electrocatalysis a robust technology for water purification.

(37) $$\text{Semiconductor}+\text{hv}\;\;\to \;\;h^{+}+e^{\text{–}}$$

(38)

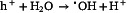

(39) $$O_{2}+e^{-}\;\to \;O_{2}^{•–}$$

(40) $$O_{2}+2e^{-}\;\to \;\;H_{2}O_{2}$$

(41)

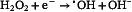

(42) $$h^{+}/O_{2}^{•\text{–}}{/}^{•}\text{OH}+\text{pollutant}\;\;\to \;\;{\text{CO}}_{2}+H_{2}O$$

It is important to note that while these reaction pathways are well established, their efficiency and selectivity are strongly influenced by the semiconductor physiochemical properties and the wastewater matrix composition [101].

Recent reviews have comprehensively described the photo‐electrochemical oxidation systems. For instance, Khan et al. systematically discussed electrode materials, reactor design, and the mechanisms governing reactive species generation, with emphasis on the degradation of dyes and pharmaceuticals [102]. They presented strategies, such as doping, heterojunction formation, and morphology control, as methods to enhance the photocurrent response, light absorption, and degradation efficiency in controlled conditions. Similarly, Issaka et al. highlighted catalyst synthesis routes, mechanistic pathways, and operational parameters as key factors affecting the degradation kinetics in photo‐electrochemical processes [103].

These works emphasize the role of material design in improving the reaction rate in a photoelectrochemical system. In addition, a review on non‐TiO_2_ photoanodes for photo‐electrochemical water treatment indicated that focusing on synthesis strategies and structural optimization can enhance the utilization of visible light and degradation performance [104]. These studies collectively provide a strong foundation for understanding photo‐electrochemical systems, particularly in terms of material development, reaction mechanisms, and performance optimization in laboratory conditions.

Recent research has focused on the development of visible‐light active semiconductors because they can use solar energy. As wide‐bandgap semiconductors may not respond to visible light due to the large energy of excitation required for the charge carrier generation, many researchers explored tuning the semiconductor bandgap or morphology toward low‐energy activation. However, the narrow bandgap of visible‐light active semiconductors facilitates the fast recombination of photogenerated holes and electrons, reducing their lifetime and limiting the degradation efficiency. Therefore, heterojunction formation, element doping, and surface modifications have been used to reduce the charge carrier recombination rate and increase their mobility and separation, as discussed in Sections 4.1 to 4.3. In conclusion, visible‐light active semiconductors are a promising option for sustainable photo‐electrocatalysis, but important issues need to be addressed to achieve high degradation efficiency [105].

### 4.1 Size‐ and Morphology‐Dependent Bandgap Tuning

The semiconductor optical features are influenced by its size and morphology, as widely reported in the literature. Previous studies consistently described morphology (e.g., nanoparticles, nanorods, nanosheets, and hierarchical structures) control as a strategy to enhance light absorption and increase surface area, thereby improving photocurrent density and degradation efficiency [104]. This review showed the relationship between morphology and performance, interpreted through intrinsic material properties, where higher surface area leads to more active sites and improved interaction with light.

Therefore, the bandgap energy can be tailored during the semiconductor production by tuning the synthesis conditions, such as temperature, solvent, and starting material [105, 106]. Indeed, the spatial confinement of electron and hole pairs vary from one dimension to another [107]. Size‐dependent bandgap tuning can improve the semiconductor properties; as the semiconductor size decreases, its energy level becomes more discrete, thus affecting the bandgap energy [108]. In general, smaller nanoparticles possess larger surface area per volumes, leading to more active sites for the interaction with the electrolyte and for enhancing photo‐electrochemical oxidation. In addition, morphology determines the spatial distribution of charge carriers and reactive species generation sites. For example, one‐dimensional nanostructures, such as nanowires, facilitate directional electron transport and reduce recombination [109]. Leelevathi et al. [110] found that the optical absorption increase in ZnO/Au nanowires, compared with ZnO/Au nanoparticles, leads to strong electronic interactions. They concluded that the nanowire geometric effects contribute to improving the semiconductor photocatalytic activity. Therefore, morphology is a key parameter for improving the photo‐electrochemical efficiency. It is important to note that the morphological design governs the interplay between charge transport and surface reaction in wastewater system.

### 4.2 Doping Semiconductors With Metals and/or Nonmetals

Doping semiconductors with metal and/or nonmetals, also known as elemental doping, has been used extensively to improve their photocatalytic and electrochemical properties. Previous reviews reported that doping introduces mid‐gap states, enhances visible‐light absorption, and improves charge carrier separation by modifying the semiconductor electronic structure [111]. Elements are used as cocatalysts for improving charge separation and optical features and increasing the charge carrier lifetime in order to enhance the degradation efficiency [112]. Metals (e.g., iron, silver, gold, nickel, copper, cobalt) and nonmetals (e.g., halogens, sulfur, nitrogen) are incorporated into the semiconductor lattice structure to modify its electronic and optical features. Such dopants can also create additional energy levels and active sites within the semiconductor bandgap structure. For instance, Ogawa et al. [113], described a strategy to improve charge separation in layered Bi_4_NbO_8_Cl and consequently its photocatalytic properties during photo‐electrooxidation of organic substances. Briefly, to decrease charge recombination in the layered Bi_4_NbO_8_Cl nanoplate, they deposited a rhodium cocatalyst at the edge. Their findings suggest that it modifies the intrinsic parallel carrier flow of layered Bi_4_NbO_8_Cl into a more favorable orthogonal flow. Other semiconductor doping strategies for improving the photo‐electrooxidation of organic pollutants have been reported [95, 114]. Overall, these studies show that elemental doping enhances the semiconductor photocatalytic and electrochemical features by improving the charge separation, extending the carrier lifetime, and optimizing the optical properties.

Notably, the role of dopants extends beyond electronic modification and directly influences reaction pathways in wastewater systems. Dopants can act as active centers for oxygen reduction reactions, facilitating the formation of intermediate species [115]. In conclusion, by understanding and linking dopant chemistry to reactive species generation and competitive reaction pathways, it becomes possible to design semiconductors that are not only efficient, but also selective and stable in realistic wastewater conditions.

### 4.3 Semiconductor Heterojunction Formation

The term heterojunction describes the combination of two or more semiconductors with different bandgap energies. The different electronic properties of the two semiconductors (e.g., Fermi level, conduction band energies, valence band energies, and work function) will influence the built‐in electric field formed at their interface [116]. Semiconductor heterojunctions are produced to preserve the photogenerated holes with the highest oxidation potential that can react directly with the pollutants or with water to produce ^•^OH for the pollutant photo‐electrooxidation [117].

Semiconductor heterojunctions can be categorized in function of their bandgap alignment type (I, II, and III), the type of semiconductor used (negative and positive), and the charge transfer route (Z‐scheme and S‐scheme) [89]. The bandgap alignment type is determined by the valence and conduction band positions. Recent studies demonstrated that type II semiconductor heterojunctions are better for efficient photogenerated charge separation [118]. Therefore, the semiconductor heterojunction capacity to enhance charge separation and improve photo‐electrocatalysis efficiency is influenced by the bandgap alignment and interfacial engineering. It has been reported that the photocatalytic properties of type I semiconductor heterojunctions can be improved by creating a defect, during synthesis or by surface modification with metals, in one of the semiconductors to increase the energy level; however, these modified type I semiconductor heterojunctions look like a type II bandgap alignment [119, 120].

**FIGURE 9:**
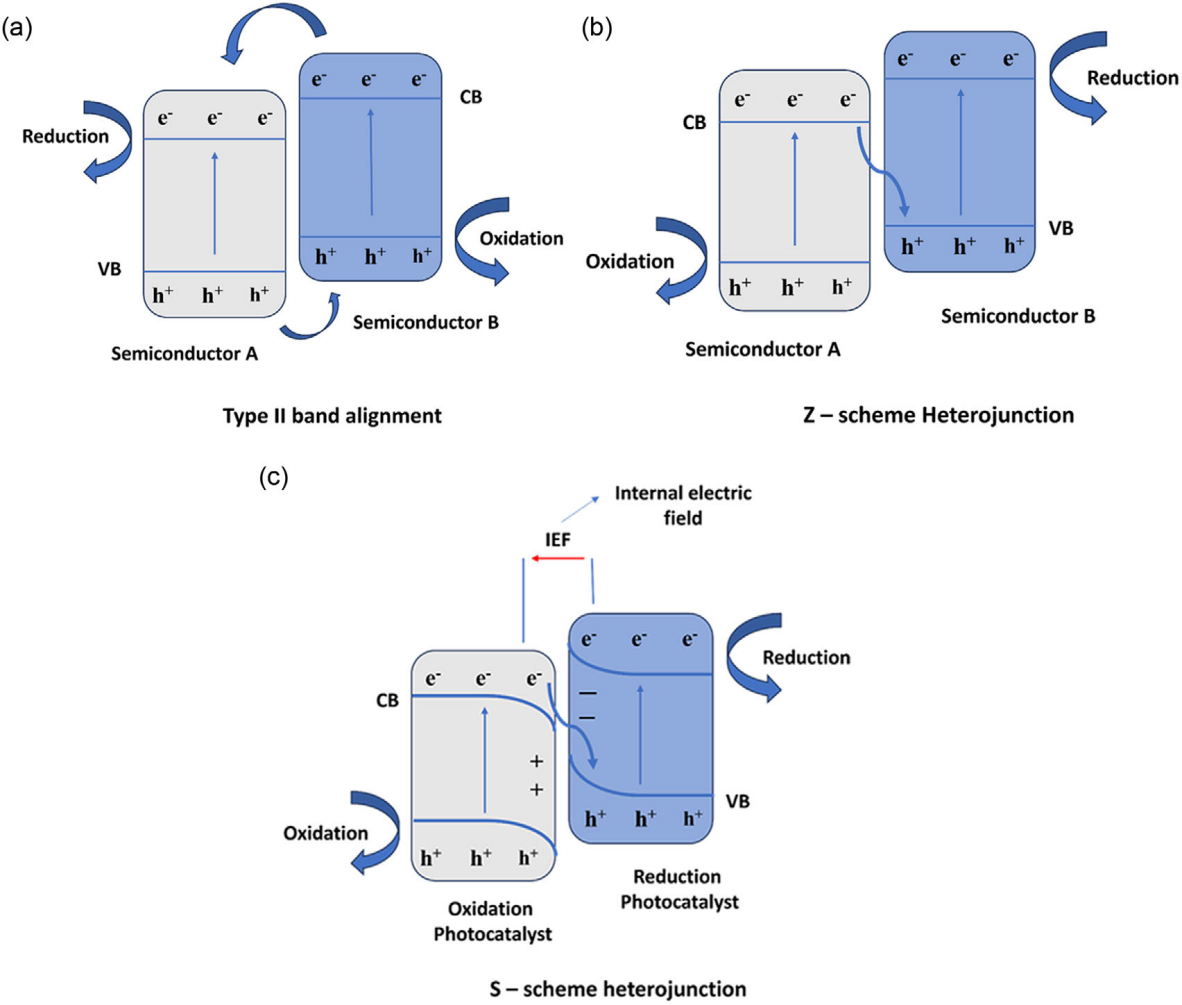
(a) Type II heterojunction. (b) Z‐ scheme semiconductor. (c) S‐scheme semiconductor. In a type II semiconductor heterojunction, the staggered band alignment can lead to fast charge carrier recombination because electrons and holes migrate to energy levels with opposite redox potentials. Therefore, Z‐scheme and S‐scheme heterojunctions have been developed to facilitate charge separation by creating internal electric fields at the semiconductor interface. Z‐scheme heterojunctions exploit electrostatic forces to facilitate effective charge recombination, while S‐scheme heterojunctions rely on the Fermi level differences between semiconductors to establish an internal electric field that improves charge carrier separation and enhances pollutant degradation in photoelectrochemical processes. Adapted with permission [121, 122]. Copyright 2022, Elsevier. *e^−^: electrons, h^+^: holes, CB: conduction band, VB: valence band.

In general, in a type II semiconductor heterojunction, the valence band of semiconductor A is more positive and has higher oxidation potential than that of semiconductor B, whereas the conduction band of semiconductor B is more negative, with higher reduction potential, than that of semiconductor A. This leads to their typical staggered‐like structure (Figure 9a) [89]. However, charge carrier recombination rate is still fast in type II semiconductor heterojunctions because upon light irradiation, holes will move from the valence band of semiconductor A to that of semiconductor B. Similarly, electrons will move from the conduction band of semiconductor B to that of semiconductor A. This is due to electron transfer from the higher to the lower Fermi level (*E*_f_). Therefore, the electrostatic repulsion between h^+^—h^+^ and e^−^—e^−^, that occurs in a type II heterojunction, leads to Z‐scheme and S‐scheme heterojunctions for photoanodes used in the photo‐electrochemical oxidation of wastewater pollutants. The major characteristic of Z‐scheme (Figure 9b) and S‐scheme (Figure 9c) heterojunctions is the formation of an internal electric field at the semiconductor interface that improves charge separation. Consequently, electrons in the conduction band of semiconductor B can interact with holes in the valence band of semiconductor A due to coulombic or electrostatic forces of attraction between these charges [121, 122]. The charge carrier separation mechanism in S‐scheme heterojunctions is due to the Fermi level differences of the two semiconductors (i.e., the oxidation and reduction photocatalysts). Upon contact, electrons of the reduction photocatalyst move to the oxidation photocatalyst until the Fermi levels reach an equilibrium. Then, an internal electric field is created at the semiconductors interface. Following electron accumulation, the band edge of the oxidation photocatalyst will bend downward and that of the reduction photocatalyst will bend upward by losing electrons. Upon light irradiation, electrons of both photocatalysts are excited from the valence to the conduction band. Due to the internal electric field, electrons generated in the oxidation photocatalyst will recombine with holes generated in the reduction photocatalyst. Moreover, the holes of the oxidation photocatalyst and electrons of the reduction photocatalyst with the highest redox potential will be retained for the oxidation and reduction reactions that lead to the production of reactive oxygen species to remove the water pollutants [122]. Overall, Z‐scheme and S‐scheme heterojunctions improve charge separation through the built‐in electric field and Fermi level equilibration. However, their efficiency is strongly influenced by the interactions at the semiconductor interface, the material stability, and the redox potential optimization.

However, these models are often applied without considering the chemical complexity of wastewater systems, where multiple redox‐active species are present. In type II heterojunctions, although charge separation is improved, the redox potential of charge carriers is reduced, which can limit the formation of highly reactive species, such as hydroxyl radicals. Conversely, Z‐scheme and S‐scheme heterojunctions preserve strong redox potential, but rely on efficient interfacial charge recombination, which can be disrupted by competing reactions in wastewater. In real wastewater systems, electrons and holes can be consumed by competing reactions, such as oxygen reduction or side reactions involving dissolved ions, thereby altering the intended function of the heterojunction formation [123]. Recent advances in photo‐electrochemical applications further demonstrate that heterojunction design can be used also to control the reaction selectivity, not just its efficiency. For example, solar‐driven photo‐electrochemical systems have been developed to convert nitrate pollutants into ammonia. In these systems, catalyst surface chemistry and interfacial charge transfer pathways suppress competing hydrogen evolution reactions and favor selective reduction. Therefore, heterojunctions studies are moving beyond band alignment models to highlight how interfacial electric fields, charge recombination pathways, and surface chemistry collectively determine degradation efficiency, mineralization and selectivity in wastewater systems [124].

### 4.4 Tandem Photo‐Electrooxidation

**FIGURE 10:**
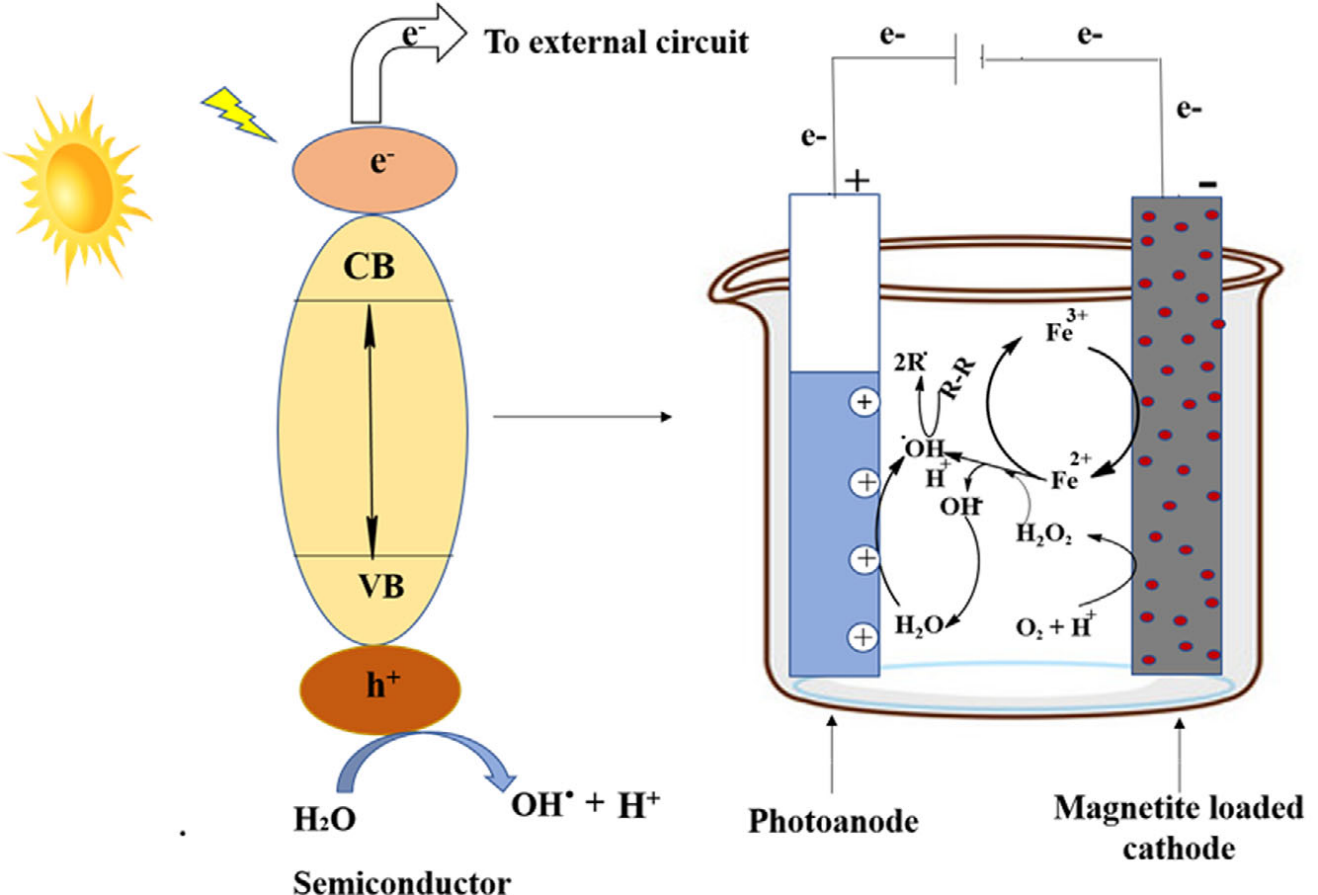
A typical photo‐electro‐Fenton set‐up in which photoelectrochemical and Fenton oxidation are combined. It features a photoelectrode, often a semiconductor (e.g., lanthanum‐doped bismuth ferrite) that generates electron–hole pairs when illuminated. These charge carriers enhance the Fenton reaction in which iron salts and hydrogen peroxide in the aqueous solution produce ^•^OH for pollutant degradation. Moreover, the counter‐electrode supports reduction reactions and the electrolyte solution facilitates ion conduction. Adapted with permission [130]. Copyright 2020, Elsevier *e^−^: electrons, h^+^: holes, CB: conduction band, VB: valence band.

Photo‐electrooxidation can be easily combined with an active photocathode and other advanced oxidation methods. For instance, a photoanode can be combined with a photocathode, giving a dual photoelectrode to reduce the energy required for pollutant degradation by photo‐electrochemical oxidation. As their combination generates an internal photovoltage, lower applied voltage is needed due to the quasi‐Fermi level differences between anode and cathode [125, 126, 127]. For instance, Orimolade and Arotiba used a Ag/BiOI photocathode and a Ag‐BiVO_4_/BiOI photoanode for the photo‐electrocatalytic degradation of diclofenac sodium [128]. Photocathodes, such as CuBi_2_O_4_ and Co_3_O_4_, have been used in dual photoelectrode‐driven photo‐electrooxidation for water treatment [125, 129]. In conclusion, dual photoelectrodes in photo‐electrooxidation reduce energy consumption and enhance charge utilization, representing a promising strategy for improving water treatment efficiency and sustainability. Other tandem photo‐electrochemical oxidation systems have been developed. For example, photo‐electrochemical oxidation and electro‐Fenton process have been combined in the photo‐electro‐Fenton process to increase the generation of reactive species [130]. In this system, a photoanode is incorporated in an electro‐Fenton system. Upon light irradiation, this allows enhanced ^•^OH production and electron transfer for water pollutant degradation (Figure 10).

Another tandem process is the combination of sonolysis with photo‐electrochemical oxidation, where ultrasound waves and light are used to increase the mineralization rate. For instance, Antonelli et al. [120], reported the degradation of ofloxacin using a Ti/Ru_0.3_Ti_0.7_O_2_ photoanode in a photo‐assisted sono‐electrochemical system. The synergistic effect of the photochemical, electrochemical and sonochemical processes allowed removing >98% of ofloxacin in 5 min and 60% of TOC after 60 min. This high efficiency can be explained by the increased ^•^OH production. However, the process scalability and energy efficiency need to be optimized because of the high energy demand of ultrasound and the potential limitations of some photoanodes. Other tandem photo‐electrochemical oxidation systems also have been explored, particularly the integration of separation technologies, such as membrane filtration and reverse osmosis [131]. In these systems, photo‐electrocatalysis facilitates the degradation and mineralization of pollutants, while the membrane unit enhances selectivity, enables retention of intermediates, and mitigates fouling, resulting in the overall improvement of the treatment efficiency. Consequently, such tandem photo‐electrochemical systems provide a robust and scalable approach for wastewater treatment, combining enhanced degradation kinetics with efficient pollutant separation and process stability.

In perspective, photo‐electrochemical oxidation for water treatment can be tailored toward sustainability when visible‐light active semiconductors are used to harness solar energy. Research is still ongoing to overcome the fast recombination rate of photogenerated holes and electrons that affects the photo‐electrochemical degradation of pollutants. Morphology tuning, doping of semiconductors and semiconductor heterojunctions have been efficiently used to overcome this challenge. The quest for novel or suitable semiconductor photoanodes and innovative cell designs should push photo‐electrooxidation as efficient on‐site, decentralized, complementary, or alternative water treatment solutions. Therefore, future research should prioritize the integration of material innovation with reactor design and process optimization, enabling the transition of photo‐electrochemical systems from laboratory studies to real‐world wastewater treatment applications.

## 5 Electrochemical Activation of Persulfates

Electrochemical advanced oxidation processes can be combined with other technologies, such as persulfate based advanced oxidation, for providing green solutions to decrease their electric energy consumption and to degrade pollutants via the generation of reactive oxygen species on the electrode surface [132, 133]. Compared with H_2_O_2_ based advanced oxidation processes, persulfate based advanced oxidation processes are: (1) less dependent on the operating parameters; (2) they produce SO_4_^•−^ that has a longer lifetime than ^•^OH; and (3) their storage and transport costs are lower [134, 135]. Peroxymonosulfate (SO_5_^2−^) and peroxydisulfate (S_2_O_8_^2−^) are the most used persulfates. Upon activation, they produce SO_4_^•−^ by energy and electron cleavage of the peroxide bonds in persulfate molecules [136].

Currently, different persulfate activation methods are used, including catalysts, carbon based catalysts, transition metal catalysts, ultraviolet light, ultrasounds, and electrochemical activation [137, 138, 139]. Extensive reviews have been published on various catalysis types and mechanisms for activating persulfates. For instance, Wang et al. discussed the interfacial catalysis and irradiation synergy of functional carbon nitride materials for peroxymonosulfate activation, with a focus on the functionalization strategies and corresponding catalytic mechanisms of both metal free and metal containing carbon nitride materials [135]. Shang et al. provided a comprehensive overview of single atom catalysts for advanced oxidation processes, systematically introducing the degradation mechanisms of micropollutants on carbon based single‐atom catalysts [140]. However, there is a lack of timely and systematic updates on the recently emerging electrochemical activation technology. This section will focus on the recent advances in electrochemical techniques for persulfate activation to address this research gap.

Electrochemical activation is environmentally friendly, requires mild operating conditions, easy operation, and low energy consumption, unlike other persulfate activation methods [141]. The electrode materials used for persulfate electrochemical activation mainly include carbon based electrodes, carbon–metal composite electrodes, and metal electrodes [142, 143, 144, 145]. Persulfates can be activated by both cathode and anode via electron transfer at the electrode surface to produce SO_4_^•−^, ^•^OH, or other reactive species (e.g., ^1^O_2_, O_2_^•−^). However, due to the molecular structure differences between peroxymonosulfate and peroxydisulfate and the difference in the mechanism of the anode and cathode electrochemical activation, the electrochemical activation pathways of peroxymonosulfate and peroxydisulfate at the cathode or anode and the organic pollutant removal mechanism need to be thoroughly studied to facilitate the application of these systems [146].

### 5.1 Anodic Activation of Persulfates

**FIGURE 11:**
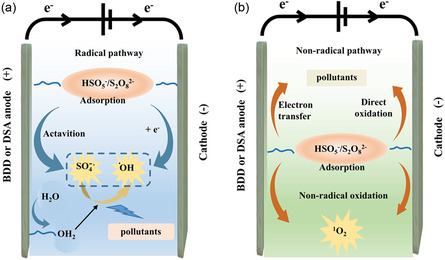
Mechanism of cathode or anode (boron‐doped diamond or dimensionally stable anode) activation of persulfate, peroxymonosulfate and peroxydisulfate to degrade pollutants. The mechanism involves two pathways that work together: (a) the radical pathway, where water and persulfate ions are activated at the anode to produce reactive radicals, ^•^OH and ⋅SO_4_^−^^•^, that degrade pollutants and (b) the nonradical pathway, where pollutants are oxidized directly or through species, such as singlet oxygen (^1^O_2_), activated at the cathode. Adapted with permission [148]. Copyright 2020, Elsevier. e: electrons, BDD: boron‐doped diamond, DSA: dimensionally stable anode.

Many recent studies investigated the anodic activation of persulfates due to the good recyclability, high efficiency, and environmental harmlessness. Dimensionally stable anodes, boron‐doped diamond anodes, and Ti/Pt anodes have been used with good contaminant removal [147, 148, 149]. In their systematic investigation on the electrochemical activation of peroxydisulfate at a Ti/Pt anode, Song et al. observed a significant synergistic effect between electrolysis and peroxydisulfate addition. They identified direct oxidation (via standalone peroxydisulfate oxidation or electrolysis alone) and nonradical oxidation as the primary pathways for pollutant degradation. The linear sweep voltammetry and chronoamperometry results suggested that electric discharge may facilitate the integration of peroxydisulfate molecules with the anode surface, forming a unique transition‐state structure responsible for the nonradical oxidation mechanism in the electrochemical peroxydisulfate activation process [147]. In addition, Ding et al. systematically investigated the enhanced pollutant removal performance during the electrochemical activation of peroxydisulfate using boron‐doped diamond and dimensionally stable anodes in different electrolyte medium, and also the respective contributions of various degradation mechanisms and pathways. They demonstrated that bisphenol A degradation was significantly affected by different electrolytes. Bisphenol A degradation kinetics followed the Cl^−^ > ClO_4_^−^ > SO_4_^2−^ order in the boron‐doped diamond anode/peroxydisulfate system, and the Cl^−^ > SO_4_^2−^ > ClO_4_^−^ order in the dimensionally stable anode/ peroxydisulfate system. Active chlorine was the main reactive oxygen species during bisphenol A removal in chloride media, and the contribution of SO_4_^2−^ to its degradation was higher in perchlorate than in chloride media [148]. In addition, direct electron transfer on the surface of boron‐doped diamond or dimensionally stable anodes contributed to bisphenol A removal (Figure 11).

The high preparation cost of dimensionally stable and boron‐doped diamond anodes is the bottleneck to their practical application. Therefore, transition metal based anodes have been proposed as a cheaper alternative. For example, Li et al. used an annular iron sheet as anode to electrochemically activate persulfate to degrade 2, 4‐dinitrophenol, with a significant synergetic effect [150]. Zhu et al. reported that a LiFe_5_O_8_ on Fe substrate (Fe@LFO) anode exhibited excellent structural stability for the electrochemical activation of persulfate [151]. Moreover, an antimony‐doped tin oxide electrode, electro‐activated persulfate system displayed high catalytic efficiency because persulfate ions were more easily adsorbed on SnO_2_ surface [152]. Similarly, persulfate electrochemical activation on a blue TiO_2_ nanotube anode allowed the efficient degradation of 2,4‐dichlorophenoxyacetic acid with much lower energy requirements (0.14 kWh m^−3^) compared with boron‐doped diamond and dimensionally stable anodes [153]. Persulfate electro‐activation using a transition metal‐based anode also is effective for removing organic contaminants, but it is not a long‐term solution because it is not environmentally friendly due to the metal ions dissolved from the anode during electrolysis [154]. Therefore, new cost‐effective and structurally stable transition metal‐based anodes are required.

### 5.2 Cathodic Activation of Persulfates

A synergistic effect was observed when activated carbon fibers were used as cathode for peroxydisulfate activation in electrolysis [155]. The activated carbon fiber cathode maintained an excellent stability even after 100 cycles because the cathode electrons inhibited the destruction of activated carbon fibers caused by chemical oxidation. Therefore, both anode and cathode could electro‐activate persulfates to oxidize contaminants. Moreover, ^•^OH, SO_4_^•−^, or O_2_^•−^ are generated in the systems, as described in Equations (43)–(45), and the electrons transferred from the power source to the electrolytic system effectively improve the mass transfer [156, 157]. Compared with graphite felt anodes, persulfate electro‐activation by graphite felt cathodes is more influenced by the electrolyte nature. Indeed, its activation is higher in sulfate than in perchlorate and nitrate media. The electrochemical system with Fe–Cu/hydrophilic graphite felt as the cathode also showed a good activation of peroxydisulfate. Moreover, the graphite felt hydrophilicity facilitated the degradation of diuron, and the high‐valent metal ions on the cathode generated electrons in situ to convert into the lower state to activate persulfate continuously, producing more SO_4_^•^^−^ and ^•^OH [158].

(43) $$O_{2}+e^{-}\;\to \;O_{2}^{•-}$$

(44) $$O_{2}^{•-}+{S_{2}O}_{8}^{2-\;}\;\to \;{\;O}_{2}{+\mathrm{SO}}_{4}^{•-}+{\mathrm{SO}}_{4}^{2-}$$

(45)

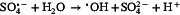




### 5.3 Anodic Activation of Peroxymonosulfate

It has been reported that electrochemically activated peroxymonosulfate tends to better remove contaminants than peroxydisulfate. Due to the presence of asymmetric peroxide bonds in peroxymonosulfate, the electric field can accelerate the electron transfer between electron donors and acceptors to promote peroxymonosulfate activation [159, 160]. Jing et al. [149] reported that the electro‐enhanced activation of peroxymonosulfate can overcome the weaknesses of electrochemical advanced oxidation processes, such as high energy consumption and pH‐dependent performance, and also the catalyst poor recyclability in these systems [161]. Indeed, carbamazepine was very efficiently removed (*k* = 0.467 min^−1^) using a very low amount of energy. Like for persulfates, both cathode and anode can electrochemically activate peroxymonosulfate, and the anodic activation of peroxymonosulfate allows the efficient treatment of emerging contaminants. Boron‐doped diamond and metal oxide anodes are frequently used [149, 150]. Yao et al. [162] proposed that the transition structure formed between peroxymonosulfate and boron‐doped diamond is the reason of the good removal efficiency and that SO_4_^•−^ and ^•^OH are the main reactive species, particularly ^•^OH. As boron‐doped diamond anodes are very expensive, metal oxide anodes have been tested to reduce costs. Zhang et al. [163] reported that the direct electron transfer efficiency and effective surface of mixed metal oxide anodes can be significantly improved when TiO_2_ nanotube array channels are uniformly filled with *β*‐PbO_2_ nanoparticles that are resistant to corrosion. This increases peroxymonosulfate activation. Alternatively, carbon‐based anodes have been widely studied due to their low cost, stable performance, and ready availability. For example, Fu et al. [164] showed that upon peroxymonosulfate activation by a carbon cloth anode, the rate constant of sulfamethoxazole degradation increased as the current density increased, possibly due to higher electron transfer. In addition, the anodic oxygen evolution at high current density hindered the adsorption of sulfamethoxazole degradation intermediates, which was conducive to maintain a clean electrode surface.

### 5.4 Cathodic Activation of Peroxymonosulfate

Peroxymonosulfate electrochemically activation by an amorphous boron cathode exhibits high selectivity for sulfamethoxazole removal with ^1^O_2_ as the main reactive oxygen species, instead of ^•^OH or SO_4_^•−^. In this system, ^1^O_2_ generation is promoted by peroxymonosulfate self decomposition induced by the interfacial alkalization and ^•^OH evolution at the cathode interface, as described in Equations (46)–(52) [165]



(46)

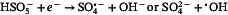






(47)

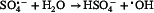






(48)

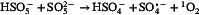

(49) $${\mathrm{HSO}}_{5}^{-}+H_{2}O{\;\to \;\text{HSO}}_{4}^{-}+H_{2}O_{2}$$

(50)

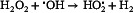

(51) $${\mathrm{HO}}_{2}^{•}\;\to \;H^{+}+O_{2}^{•-}$$

(52)

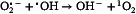




Yao et al. [166] used a mixed metal oxide electrode to electrochemically activate peroxymonosulfate for sulfadiazine degradation, and showed that both mixed metal oxide anode and cathode were implicated in this process. Sulfadiazine degradation was mediated mainly by the ^•^OH, SO_4_^•−^, and ^1^O_2_ that were obtained following peroxymonosulfate electrochemical activation (Equations (53) and (54)), the electric field excitation of oxygen to produce ^1^O_2_ (Equation (55)), and the electron transfer of adsorbed water to generate adsorbed‐^•^OH (Equation (56)). Moreover, O_2_ captured electrons on the cathode and converted to O_2_^•−^ that reacted with peroxymonosulfate to produce SO_4_^•−^ (Equations (57) and (58)).



(53)

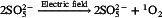






(54)

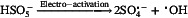






(55)

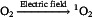






(56)

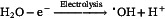

(57) $$O_{2}+e^{-}{\;\to \;\;O}_{2}^{•-}$$

(58) $$O_{2}^{•-}+{\mathrm{HSO}}_{5}^{-}\;\to \;{\;O}_{2}+{\mathrm{SO}}_{4}^{•-}+{\mathrm{OH}}^{-}$$

**FIGURE 12:**
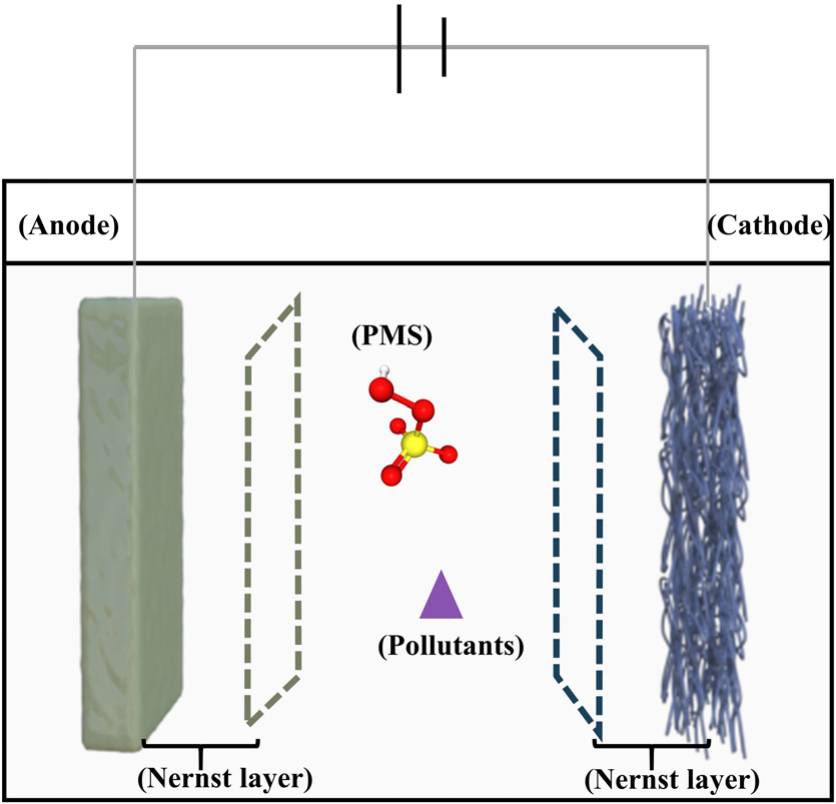
The electrode thick diffusion boundary layer in a conventional flow‐by configuration where peroxymonosulfate is electrochemically activated for pollutant removal. It highlights that diffusion plays a key role in improving the reaction control and efficiency. *PMS: peroxymonosulfate. Adapted with permission [167]. Copyright 2019, American Chemical Society.

Electro‐enhanced peroxymonosulfate activation systems are mostly developed in a conventional flow‐by configuration, i.e*.*, convection transmission parallel to the electrode surface (Figure 12). In this configuration, peroxymonosulfate activation and contaminant degradation are influenced by the thickness of the electrode diffusion boundary layer (Nernst layer, ∼100 μm) [167]. Therefore, the mass transfer of pollutants to the electrodes and the limited active surface area are usually the rate‐limiting step in electrochemical advanced oxidation processes.

**FIGURE 13:**
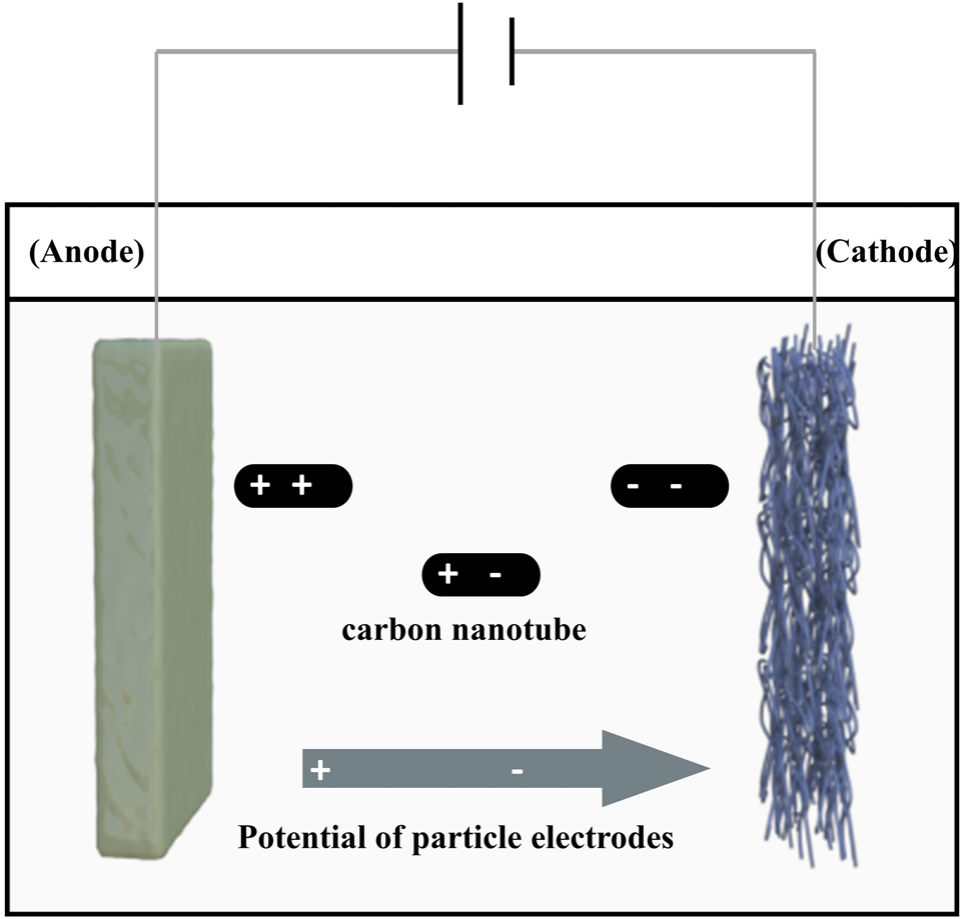
Three‐dimensional electrochemical advanced oxidation system in which particle electrodes improve the mass transfer. Localized electric fields can also induce the carbon nanotube spontaneous polarization that influences the persulfate activation pathway, and thus a more selective degradation of pollutants. Adapted with permission [167, 168]. Copyright 2019, American Chemical Society and 2013 Elsevier.

Recently, three‐dimensional electrochemical reaction systems have been developed to improve the synergy between the electric field and the catalyst and to maximize mass transfer. In these systems, heterogeneous catalysts (e.g., carbon nanotubes) are introduced in the conventional electrolysis set‐up (Figure 13) [168, 169]. The electric field gradient between anode and cathode leads to the spontaneous polarization into particle electrodes of the solid particles dispersed in the solution, reducing the cell potential and redox reactions.

The three‐dimensional electrochemical advanced oxidation system with carbon nanotubes as particle electrodes allows describing the electron transfer process for pollutant oxidation [169]. In this system, carbon nanotubes‐particle electrodes display different excited potentials (*E*_CNT‐PE_) as a function of their anode–cathode distance (Figure 13). Organic pollutants are degraded by the carbon nanotubes–peroxydisulfate complexes generated at the surface through the electron‐transfer process when the *E*_CNT‐PE_ value is greater than the water oxidation potential and lower than the oxidation potential of the organic pollutant, but not when the *E*_CNT‐PE_ is lower than the pollutant oxidation potential. In addition, when the *E*_CNT‐PE_ is higher than the oxidation potentials of water and pollutant, surface‐bound radicals are generated and they can rapidly degrade organic pollutants and produce hydroxylated products to maintain the carbon nanotube ‐ particle electrode surface clean, which allows the microelectrodes to be used for a longer period of time.

Table 3 summarizes recent advances in the degradation of organic compounds using electrochemical advanced oxidation process‐persulfate/peroxymonosulfate systems. Several materials have been tested as particle electrodes for persulfate electro‐activation, such as MnFe_2_O_4_, CoFe_2_O_4_, activated carbon, activated carbon loaded with metallic Co, and sulfur‐doped carbon. Conversely, the mechanisms underlying the organic pollutant degradation and the electroreaction pathway of persulfate activation need to be better elucidated [150, 163, 174, 175, 176]. Unfortunately, the microelectrode polarization potential has not been precisely measured yet, and the nonradical oxidation pathways for persulfate electrochemical activation need more investigations [169].

**TABLE 3:** Electrochemical advanced oxidation process‐persulfate/peroxymonosulfate systems to degrade organic pollutants. This table outlines various electrochemical systems for degrading pollutants using anode and cathode electro‐activation of peroxymonosulfate and persulfate. It features different electrode materials, including *β*‐PbO_2_/Ti, carbon cloth, and Pt‐plated titanium, with degradation efficiencies ranging from 80% to 100%, depending on the pollutant and reaction conditions. Most systems demonstrate high efficiency, often >90%.

System		Electrode	Pollutant	Reaction conditions	Degradation efficiency, %	Reference
Anode electro‐activation of peroxymonosulfate		A: β‐PbO_2_/TNAs/Ti anode C: Anatase TNAs/Ti cathode	Berberine	C_0_ = 0.1 M, [peroxymonosulfate] = 10 mM, *j* = 17.5 mA cm^−2^, NaNO_3_ = 50 mM, pH = 6.9, reaction time = 180 min	100%	[163]
		A: Carbon cloth anode C: Anatase TNAs/Ti cathode	Sulfamethoxazole	C_0_ = 10 μM, [peroxymonosulfate] = 1 mM, *j* = 5 mA cm^−2^, [Na_2_SO_4_] = 50 mM, pH = 3.6, reaction time = 30 min.	80%	[164]
		A: Ti/D‐Ce/Sb_2_O_3_ anode C: Ti plate cathode	Perfluorooctane sulfonate	C_0_ = 10 mg L^−1^, [peroxymonosulfate] = 5 mM, *j* = 10 mA cm^−2^, reaction time = 30 min.	93.2%	[170]
Cathode electro‐activation of peroxymonosulfate		A: Pt‐plated titanium anode C: Activated carbon fiber cathode	Carbamazepine	C_0_ = 0.042 mM, [peroxymonosulfate] = 50 mM, *j* = 28.6 mA cm^−2^, pH = 3, reaction time = 60 min.	96.39%	[160]
		A: Palladium ceramic membrane anode C: Palladium ceramic membrane cathode	Methylene blue	C_0_ = 10 μM, [peroxymonosulfate] = 0.1 mM, transmembrane pressure = 0.1 bar, filtration volume = 50 mL, pH = 5.7; DC supply = 1.6 V;	94.50%	[171]
Anode electro‐activation of persulfate	A: Blue‐TiO_2_ nanotube anode C: Stainless steel cathode		Phenol	C_0_ = 100 mg L^−1^, *j* = 2.5 mA cm^−2^, pH = 5, [Na_2_SO_4_] = 50 mM, reaction time = 240 min.	87%	[143]
	A: PTFE‐carbon anode C: Stainless steel cathode		Sulfamethoxazole	C_0_ = 5 μM, *j* = 10 mA cm^−2^, pH = 5, [NaClO_4_] = 50 mM, reaction time = 5 min.	100%	[172]
Cathode electro‐activation of persulfate	A: Platinum sheet anode C: Fe–Cu graphite felt cathode		Diuron	C_0_ = 20 mg L^−1^, [persulfate] = 1 mM, *j* = 2.5 mA cm^−2^, pH = 5.7, [Na_2_SO_4_] = 50 mM, reaction time = 60 min.	100%	[158]
	A: Platinum sheet anode C: Biochar catalytic cathodes		Sulfamethazine	C_0_ = 40 mg L^−1^, [persulfate] = 10 mM, [catalyst] = 1 g L^−1^, I = 50 mA, reaction time = 60 min.	95%	[157]
Three‐dimensional discrete electrodes	A: Graphite plate anode C: Graphite plate cathodes Catalysts: Carbon nanotubes		2,4‐dichlorophenol	C_0_ = 0.5 mM, [persulfate] = 1 mM, [CNT] = 0.1 g L^−1^, *j* = 1.0 mA cm^−2^, pH = 6.0, [Na_2_SO_4_] = 50 mM,	98.2%	[169]
	A: Ti/RuO_2_‐IrO_2_ anode C: Ti mesh cathodes Catalysts: N‐doped activated carbon		4‐chlorophenol	C_0_ = 80 mg L^−1^, [persulfate] = 5 mM, [catalysts] = 0.1 g L^−1^, *j* = 2.0 mA cm^−2^, pH = 5.3, [Na_2_SO_4_] = 20 mM,	98.3%	[173]

Abbreviation: 4‐CP: 4‐chlorophenol, C_0_: initial concentration, CNT: carbon nanotube, PTFE: polytetrafluoroethylene, TNAs: titanium dioxide nanotube arrays.

**FIGURE 14:**
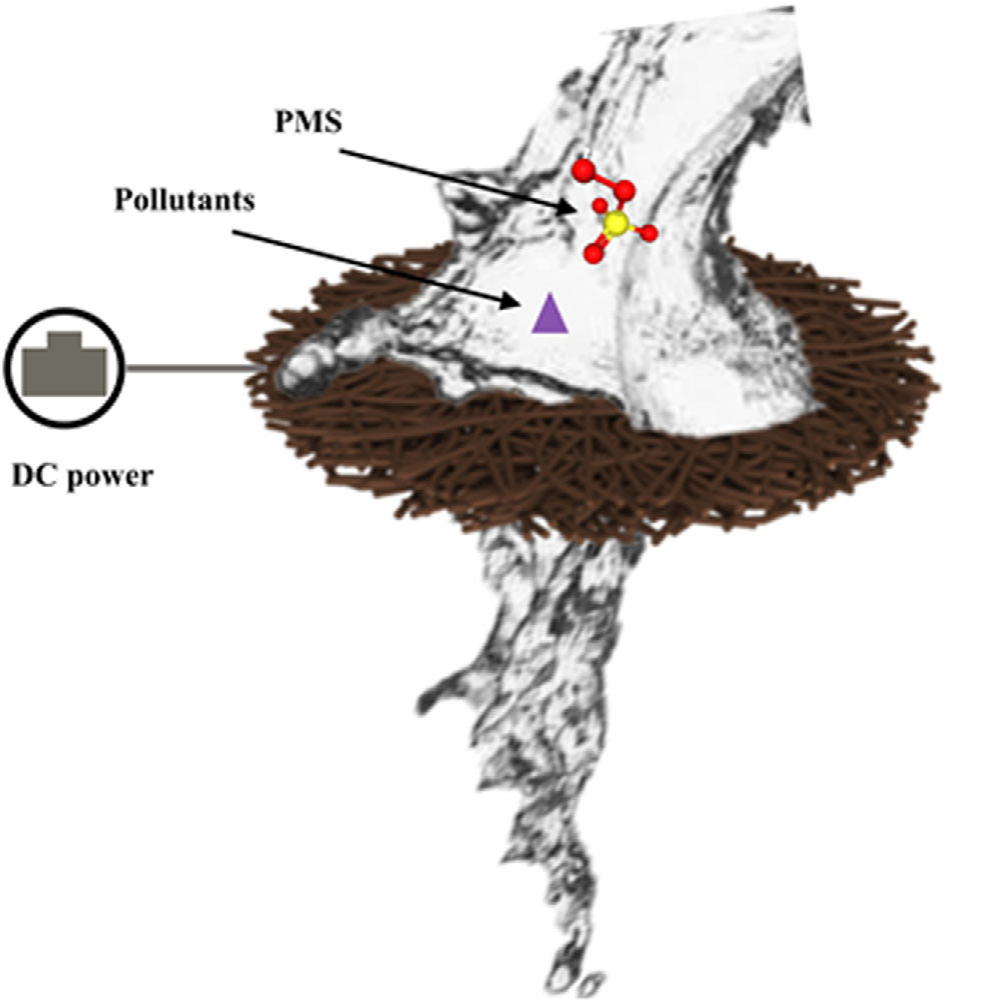
Diagram of an electroactive filtration device. The image emphasizes the simplicity of filtered electrodes in enhancing mass transfer and effluent treatment efficiency through the cathode–anode synergism that also reduces energy consumption. This system helps to overcome the recovery difficulties of granular electrodes and reduces metal leaching. Adapted with permission [167, 177]. Copyright 2019, American Chemical Society and 2012, American Chemical Society. DC: direct current, PMS: peroxymonosulfate.

A flow‐through configuration (i.e., convective transmission perpendicular to the electrode surface) can be obtained using an electrical filtration device based on membrane electrodes [167, 177] (Figure 14). This configuration can greatly increase charge transfer and mass transfer on the electrode surface [160]. For example, Zhao et al. [171] developed an electrified ceramic membrane filtration system to degrade micropollutants through peroxymonosulfate electro‐activation. They showed that the enhanced convective mass transfer and spatial confinement boosted peroxymonosulfate electro‐activation and significantly reduced energy consumption. According to the authors, the ultra‐efficient micropollutant removal was due to ^1^O_2_ intensification and direct electron transfer from micropollutants to peroxymonosulfate. Moreover, Jin et al. [178] showed that sulfamethoxazole can be efficiently degraded using an electrified filter functionalized with single Fe atoms. In this system, the intrinsic activity of single atomic sites was improved by applying an external electric field to facilitate the transition metal circulation. The Fe single atom sites were responsible for peroxymonosulfate adsorption and activation to generate SO_4_^•−^ upon application of the electric field.

## 6 Computational Chemistry for Organic Pollutant Electrooxidation

As discussed in the previous sections, the electrode material and other operational parameters are crucial for determining the degradation efficacy, current efficiency, and treatment type. The anodic material role is mainly explained by its influence on the involved chemical and electrochemical mechanisms that are related to the direct or indirect interactions of the organic or inorganic compounds with the electrode surface.

A comprehensive range of electrodes has been extensively tested for degrading and mineralizing different organic compounds in synthetic and real water matrices [179, 180, 181]. These electrodes are mainly classified in active and nonactive, in function of their capacity to produce free hydroxyl radicals. Nonactive anodes display very high effectiveness in electrooxidation processes because of their elevated overpotential for O_2_ evolution and enhanced capacity to generate ^•^OH [19]. However, other oxidants can be generated through chemical or electrochemical means using other anode types [15]. Therefore, the choice of anode preparation method significantly influences the oxidant performance, stability, electrochemical activity, and selectivity.

The water matrix composition also must be taken into account when determining the appropriate oxidant to be generated. Depending on the oxidant precursors, a complex behavior can be observed in some electrocatalytic materials that can result in comparable degradation/mineralization efficiencies with both active and nonactive anodes. Therefore, it is imperative to understand the surface mechanisms at the electrochemical double layer that facilitate the oxidant production or enhance the electrooxidation of organic compounds [15].

In recent years, computational chemistry has received great attention for understanding the electrooxidation mechanisms in environmental contexts and for developing novel electrocatalytic materials. From a computational point of view, the reactions and interactions that take place at the interface between the electrode and the solution, commonly referred to as the “electrical double layer,” “reaction cage,” or “Nernst layer,” are of vital importance for understanding the electrooxidation process [15]. However, the literature offers only general insights into the oxidation routes. Currently, little is known about the effects of the reaction cage characteristics on the activity and selectivity of electrochemical processes and about the interface dimensions and the active zone where oxidants are produced and react [15]. Gaining knowledge on these processes could facilitate the understanding of the formation and reactivity of oxidizing species [182, 183].

The oxidant formation and stability at the anodic surface are closely linked to the Nernst layer. Yet, comprehensive data are lacking on the basic behaviors of all oxidizing species and this hinders our understanding of the mechanisms involved in their generation, decomposition, activation, and implication in organic pollutant degradation. Computational chemistry has helped to understand the Nernst layer role in the electrocatalysis for wastewater treatment and consequently the involved mechanisms and reaction pathways [15, 182, 183, 184].

These theoretical approaches have facilitated the simulation of the electrode/solution interface to model different mechanisms at the surface‐layer or volume‐solution level. By incorporating the electrochemical interface structure, significant advancements can be made in modeling the interactions between species in solution (adsorbed and nonadsorbed) and the electrode surface by using theoretical and computational strategies [184, 185]. Indeed, theoretical and computational methods can contribute to tackle many unresolved fundamental questions about the electrochemical and chemical reactions. They can also facilitate the translation of technological advancements from laboratory settings to practical applications at different scales. Therefore, these tools could open new opportunities for applied electrochemistry in the environment.

**FIGURE 15:**
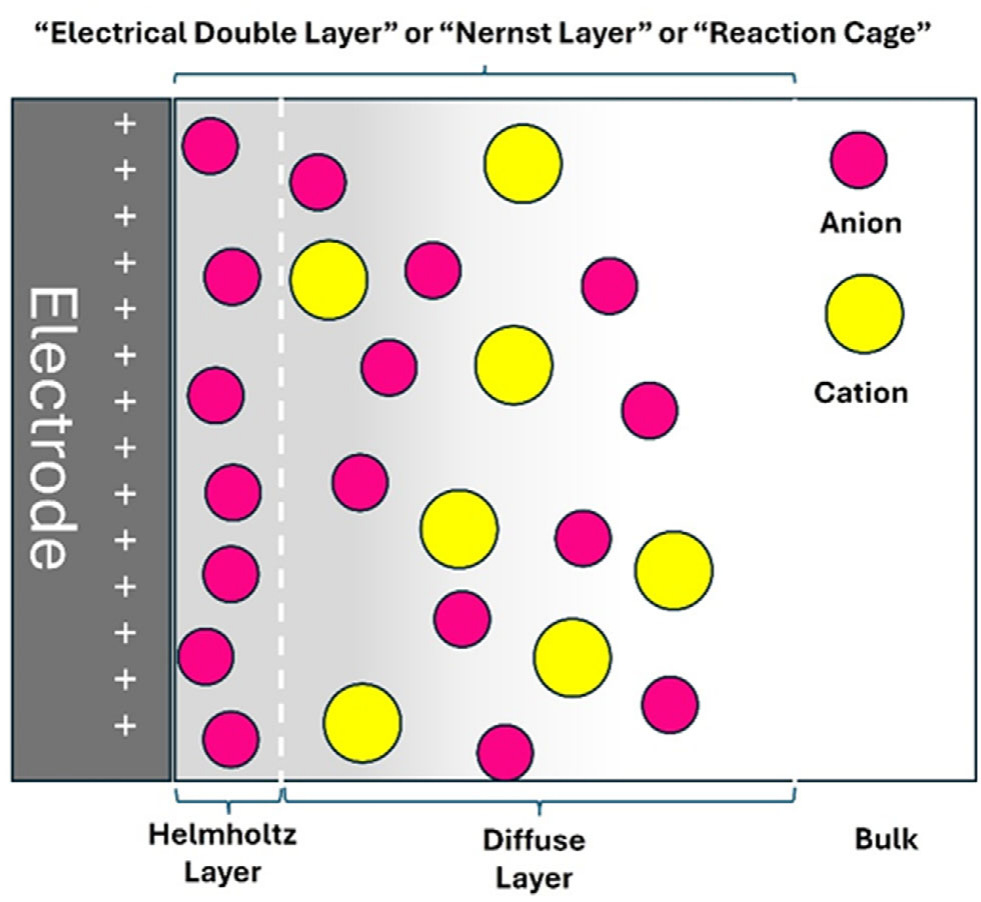
The electrical double layer, also called reaction cage or Nernst layer, is the interface between the electrode and electrolyte solution. Anions/cations arrange themselves near the electrode surface when a potential is applied. The Helmholtz layer has two planes, inner and outer. In the inner plane, ions are tightly bound to the electrode, whereas in the outer layer, solvated ions are more loosely held. Currently, theoretical and computational approaches are used to understand the electrochemical processes as a fundamental part of all applications of environmental electrochemistry. Adapted with permission [184]. Copyright 2018, Elsevier.

Theoretical and computational chemistry are remarkably effective tools for investigating homogeneous and heterogeneous reactions in electrochemistry. These approaches employ first principles or empirical methods to predict transformation behaviors and to analyze the double layer structure and features in electrochemical systems [184, 186]. In recent years, computational approaches have been used to develop and optimize electrooxidation‐based technologies by modeling the water matrix/solution at the electrode–electrolyte interface (Figure 15) [184].

Microscopically, the inclusion of electrolytes or ionic species in intricate water matrices renders instrumental analysis approaches inadequate for investigating the reaction cage structure. This is even more obvious in the case of intricate matrices in which organic or inorganic pollutants, reactive oxidizing species, radical or nonradical, and natural organic matter coexist [15]. In this context, computational methodologies are appealing due to their use of simulations, modeling, theoretical analyses and computational calculations.

The most important aspects that can be investigated using theoretical and computational approaches are [187]:

i. The solvent behavior in relation to the applied electrochemical potential;
ii. The effect of current‐induced interfacial concentration gradients on the electrochemical kinetics;
iii. The effect of cations that are nonspecifically adsorbed onto the outer Helmholtz plane on the reaction selectivity and activity;
iv. The electrode–pollutant interactions;
v. The influence of the anode electronic structure on the dynamic conformational interactions;
vi. The possible multipoint interactions between organic pollutants, oxidants and anodic surface;
vii. The mechanisms underlying volume‐based chemical oxidation reactions involving oxidants, mediators and pollutants;
viii. The presence of adsorbed and nonadsorbed species on the anode surface.

Significant progress has been made in correlating the computational and experimental data on these research topics in order to understand electrooxidation at the atomic level [15, 184]. Nevertheless, significant theoretical hurdles still remain when studying electrooxidation at the electrode–electrolyte interface [15]. The available studies exploited mainly quantum mechanics, ab initio and molecular mechanics, empirical computing approaches. Quantum mechanics focuses on theoretical chemistry, such as post‐Hartree‐Fock and Hartree‐Fock methods, and relies on software tools, particularly those from the Gaussian series [15, 184]. This helps to accurately predict the electronic properties, molecular structure and reaction mechanisms in chemical processes that involve electrons [15]. When combined with conformational analysis and molecular dynamics, quantum mechanics calculations allow understanding environmentally assisted oxidative processes at the molecular scale and the mechanisms implicated in organic pollutant degradation, byproduct formation and specific oxidant production while avoiding the generation of toxic species. Conversely, molecular mechanics mainly models biological systems and assembly of materials that consist of thousands to millions of atoms. Simpler mathematical descriptions of the systems are employed, often omitting electrons [15].

The most significant theoretical investigations have focused on the enigmatic nonactive nature of the diamond electrode [183]. Pioneering studies examined the electronic configuration of boron‐doped diamond using DFT calculations and determined the energies associated with interfacial processes that involve water adsorption and ^•^OH formation [188, 189, 190, 191]. The diamond surface has been modeled to gain insights into the impact of boron doping, film thickness, sp^2^‐impurities, and roughness on the surface reactivity. However, the “reaction cage” concept (electrode–electrolyte interface) requires further elaboration through specific computational investigations, such as DFT studies on boron‐doped diamond anodes. At the moment, theoretical assertions concerning this interface lack corresponding experimental validation and remain unverified. For example, two main limitations are the lack of micrometric‐scale surface models and the possibility of only one place for boron within the crystal lattice. The analysis of the energies associated with interfacial interactions that involve water and ^•^OH adsorption provided some insights into the potential impact of the experimental conditions on ^•^OH production on a boron‐doped diamond electrode [191]. To this aim, the use of vacuum system modeling yielded valuable insights into the interactions between boron and adsorbates that are crucial to understand boron‐doped diamond catalytic activity. In this study, H‐terminated diamond surfaces were employed for all calculations due to their ability to accurately depict the experimental structures of recently synthesized materials [192]. As these surfaces exhibit greater reproducibility in heterogeneous reactions that involve charge transfer, compared with oxygen termination, the thermodynamic parameters of interfacial interactions at the boron‐doped diamond surface could be determined using theoretical calculations [193].

Theoretical investigations also focused on the structure featuring boron in tetrahedral coordination, C‐sp^3^ hybridization, and H‐saturated boron‐doped diamond surfaces. This allowed understanding how to increase the mineralization of organic compounds. For example, Jaimes and coworkers successfully employed ab initio modeling to expand surface models to characterize the boron‐doped diamond properties [194]. The analysis of the adsorption behaviors of H_2_O and hydroxyl radicals in various initial positions, considering Van der Waals interactions, allowed confirming, using thermodynamic data obtained by computational calculations, the reactivity of boron‐doped diamond toward the indirect electrooxidation of organic compounds.

Another feature related to the “reaction cage” concept is the impact of dopants on the diamond surface into the production of oxidants. An interesting study considered the reaction as an outer‐sphere process and predicted the effects of nitrogen, phosphorous, sulfur and fluorine dopants on the diamond surface using DFT calculations. It also provided detailed insights into the physical and chemical aspects, specifically focusing on the free energy of adsorbed oxygen intermediates (OH*, O*, and HOO*) and the maximum Δ*G* of each oxidation reaction step [195]. These data are crucial prerequisites for understanding ^•^OH production at the anodic surface. Based on these theoretical data, the authors concluded that incorporating fluorine, sulfur, and phosphorus into the diamond film increases the Faradic efficiency for ^•^OH production. Conversely, nitrogen incorporation enhances the oxygen evolution reaction, discouraging its application in electrochemical advanced oxidation processes.

Additionally, computational calculations were also employed to investigate persulfate electrosynthesis in the context of oxidants [196]. The presence of S_2_O_8_^2−^ is facilitated by the generation of SO_4_^•−^ as the primary intermediate. Davis et al. [196], conducted both experimental and theoretical investigations using DFT and found that S_2_O_8_^2−^ is formed on a boron‐doped diamond surface with higher graphite content, where sp^2^ impurities are used as active sites. This process follows a two‐step mechanism, although the involvement of ^•^OH in SO_4_•^−^ formation was also taken into account.

Currently, many electrochemical and theoretical studies are carried out to elucidate the observed experimental phenomena related to the molecular structures and electronic properties of pollutants and anodic surfaces [195, 197, 198, 199, 200]. Their aim is to determine the optimized geometric structures and frontier orbitals through computational methods to facilitate the assessment of potential atomic sites where the highest energetic electrons are concentrated in frontier orbitals. These sites can interact with pollutants on the anodic surface through direct oxidation. DFT‐based modeling allows describing the energy barriers that must be overcome to convert pollutants into transition states, before their complete oxidation. Moreover, DFT and the quantum theory of atoms in molecules are exploited to understand electrochemical and chemical reactions at the molecular scale. This is achieved by identifying critical points where the electron density distribution is predominantly concentrated within a specific plane of the chemical bond, exhibiting a symmetrical and cylindrical distribution pattern.

Byproduct generation during electrooxidation of organic pollutants on a boron‐doped diamond surface can be predicted by combining experimental data and simulations. The analysis revealed, supported by the examination of the highest occupied molecular orbital energies, that compounds undergo oxidation through a direct electron transfer mechanism, facilitated by ^•^OH [197]. Another study compared a three‐dimensional SnO_2_‐Sb anode with macropores and a boron‐doped diamond electrode for the electrooxidation of ciprofloxacin [201]. The intermediates were identified using analytical methods, and DFT calculations helped to demonstrate that the anode surface electronic characteristics, its proximity to the solution.

When using theoretical and computational tools for electrochemistry studies, their advantages (i–iv) and disadvantages (v–viii) must be considered, particularly [15, 184]:

i. They allow obtaining reasonable approximations;
ii. They reduce the number of experiments and the associated risks;
iii. Their predictions are in line with the experimental data/expectations;
iv. They allow modeling molecular‐level reactions to facilitate anode development;
v. They cannot accurately describe intermolecular interactions;
vi. Users must have sufficient experience in computational methods to select the appropriate techniques;
vii. Users must have enough experience and knowledge for selecting the most suitable theory level, such as functional, basis set, and solvation model, for a specific calculation objective;
viii. Comparisons and checks must be carried out with other calculation methods and the existing literature data to minimize inaccurate conclusions or misunderstandings.

In the near future, interactions between chemists and physicists with strong skills in such sophisticated computational tools and concepts should be promoted. It will be also important to understand how the electronic structure of the electrocatalytic materials influences the interactions between organic pollutants and electrode surfaces and the multipoint interactions [15]. In addition, future studies should focus on water matrices and solvation effects and also on the oxidant interactions at the surface layer or in the bulk solution. Ultimately, it is crucial to emphasize that the correlation between experimental and theoretical data has facilitated the validation of the initial electrooxidation mechanisms and the identification of new oxidation routes influenced by distinct conditions.

## 7 Conclusions

In recent years, electrochemical advanced oxidation processes have emerged as sustainable solutions for the efficient treatment of water containing a wide range of organic pollutants. The integration of electrochemical principles with advanced oxidation processes represents a breakthrough in sustainable water treatment technologies. However, research and development efforts to optimize electrode materials, operational parameters, and process integration are essential for realizing the full potential of electrochemical advanced oxidation processes as sustainable and effective solutions.

Future research should aim at (i) widening the basic knowledge, (ii) advancing technological developments for practical applications, and (iii) evaluating their cost and environmental impact in the context of water treatment. First, efforts should concentrate on better understanding the mechanisms underpinning electrochemical advanced oxidation process efficacy. Second, electrodes used in electrochemical advanced oxidation processes must be physically and electrochemically characterized to establish clear structure–function relationships. Third, the development of cost‐effective electrodes with robust performance in different operational conditions should be prioritized. In addition, the environmental impacts and operational costs of electrochemical advanced oxidation processes need to be reduced to promote the adoption of such technologies by engineers and decision makers. Furthermore, studies should also explore how to recover energy from cathodic reactions, such as hydrogen production and carbon dioxide reduction. By addressing these key issues, future research can significantly contribute to push electrochemical advanced oxidation processes as effective and environmentally sustainable solutions for water treatment. In this context, the integration of artificial intelligence (AI) offers a particularly attractive and powerful avenue. AI driven tools can be leveraged to accelerate the discovery and design of high‐performance electrode materials through data‐driven screening and predictive modeling, enabling the identification of optimal compositions and structures with enhanced catalytic activity and stability. In parallel, machine learning algorithms can be used to model complex reaction pathways and operational parameters, allowing the real‐time optimization of electrochemical advanced oxidation process performance with different water matrices. AI can also support process scale‐up by predicting energy consumption, treatment efficiency and byproduct formation, thereby improving both economic and environmental sustainability.

## Author Contributions

**Fida Tanos**: conceptualization, writing – original draft, methodology, validation, visualization. **Chaimaa Gomri**: conceptualization, methodology, validation, writing – original draft. **Clément Trellu**: conceptualization, writing – original draft, methodology, validation. **Mehmet A. Oturan**: conceptualization, writing – review & editing, supervision, validation. **Shuaishuai Li**: conceptualization, writing – review & editing, validation, visualization, writing – original draft. **Minghua Zhou**: writing – review & editing, supervision, conceptualization, validation. **Kehinde D. Jayeola**: conceptualization, writing – original draft, validation, investigation, visualization. **Omotayo A. Arotiba**: conceptualization, visualization, writing – review & editing, supervision, funding acquisition. **Elisama Vieira dos Santos**: writing – original draft, conceptualization, methodology, validation, visualization. **Carlos A. Martínez‐Huitle**: conceptualization, supervision, methodology, validation, investigation, visualization, writing – review & editing. **Mikhael Bechelany**: writing – review & editing, funding acquisition, conceptualization, methodology, validation, supervision, project administration. **Marc Cretin**: writing – review & editing, conceptualization, formal analysis, supervision, validation, visualization.

## Conflicts of Interest

The authors declare no conflicts of interest.

## 8 Declaration

MAO declares that he is Editor of Environmental Chemistry Letters.

## Data Availability

The data that support the findings of this study are available from the corresponding author upon reasonable request.
